# The 1-Particle-per-k-Nucleotides (1PkN) Elastic Network Model of DNA Dynamics with Sequence-Dependent Geometry

**DOI:** 10.3389/fphys.2017.00103

**Published:** 2017-03-14

**Authors:** Takeru Kameda, Shuhei Isami, Yuichi Togashi, Hiraku Nishimori, Naoaki Sakamoto, Akinori Awazu

**Affiliations:** ^1^Department of Mathematical and Life Sciences, Hiroshima UniversityHiroshima, Japan; ^2^Research Center for the Mathematics on Chromatin Live Dynamics, Hiroshima UniversityHiroshima, Japan

**Keywords:** double-stranded DNA, sequence-dependent geometry, coarse-grained, elastic network model, normal-mode analysis, mitochondrial DNA

## Abstract

Coarse-grained models of DNA have made important contributions to the determination of the physical properties of genomic DNA, working as a molecular machine for gene regulation. In this study, to analyze the global dynamics of long DNA sequences with consideration of sequence-dependent geometry, we propose elastic network models of DNA where each particle represents *k* nucleotides (1-particle-per-k-nucleotides, 1PkN). The models were adjusted according to profiles of the anisotropic fluctuations obtained from our previous 1-particle-per-1-nucleotide (1P1N) model, which was proven to reproduce such profiles of all-atom models. We confirmed that the 1P3N and 1P4N models are suitable for the analysis of detailed dynamics such as local twisting motion. The models are intended for the analysis of large structures, e.g., 10-nm fibers in the nucleus, and nucleoids of mitochondrial or phage DNA at low computational costs. As an example, we surveyed the physical characteristics of the whole mitochondrial human and *Plasmodium falciparum* genomes.

## Introduction

Genomic DNA plays many functional roles such as a medium for the storage of genetic information and as a molecular machine involved in gene expression regulation. A recent genome-wide analysis of the intra-nuclear chromosomes of eukaryotes suggested that not only chemical but also sequence-dependent physical properties of DNA predominantly contribute to formation of the genomic structure and gene expression regulation (Shrader and Crothers, 1989; Cacchione et al., 1997; Lowary and Widom, 1998; Widlund et al., 1999; Geggier and Vologodskii, 2010). Specifically, sequence-dependent structures play a key role in the determination of functional properties such as nucleosome positioning in DNA (Freeman et al., 2014b; Awazu, 2017).

Genomic DNA in prokaryotes, viruses, and mitochondria forms compact structures called nucleoids. The structures are shaped through the bending and twisting of DNA, mediated by histone-like proteins such as mitochondrial transcription factor A (TFAM) (Alam et al., 2003; Pohjoismäki et al., 2006; Kaufman et al., 2007; Ngo et al., 2014) and HU proteins (Broyles and Pettijohn, 1986; Kobryn et al., 1999; Guo and Adhya, 2007), with the help of topoisomerases (Brown et al., 1979; Wasserman and Cozzarelli, 1991; Nenortas et al., 1998; Wang, 2002). Each nucleoid generally contains negative supercoils of DNA, which notably enhance the transcription and replication of genetic loci (Bogenhagen et al., 2014; Farge et al., 2014; Kukat et al., 2015). This observation indicates that the physical processes involving DNA, such as the formation and deformation of nucleoids, perform important functions in the regulation of gene expression in prokaryotes.

Coarse-grained (CG) models of DNA have facilitated investigations of the dynamics of relatively large DNA molecules for long time scales at reduced computational costs. Recently proposed CG models can accurately reproduce some of the properties observed in experiments (Freeman et al., 2014a; Hinckley and de Pablo, 2015; Markegard et al., 2015; Snodin et al., 2015; Singh and Granek, 2016) or all-atom models (Savelyev and Papoian, 2010; Setny and Zacharias, 2013; Naômé et al., 2014), which also depend on solution conditions. However, due to the large degrees of freedom, the computational cost of these models is still not low enough to be applicable to long DNA sequences containing more than 10^4^ base pairs, corresponding to a 10-nm fiber with 100 nucleosomes or a mitochondrial nucleoid.

Recently, we devised a simple CG elastic network model, in which each nucleotide is described by one particle, named the 1-particle-per-1-nucleotide (1P1N) model. This model incorporates a sequence-dependent basic structure, and can reproduce the dynamic behavior around the basic structure observed in all-atom models (Isami et al., 2015). In this paper, to further reduce the computational cost, we propose a 1-particle-per-k-nucleotides (1PkN) model, i.e., a CG elastic network model where one particle represents *k* nucleotides. To construct feasible 1PkN models for *k* > 1, we designed interaction networks to reproduce the behavior of the 1P1N model, and determined appropriate *k* values. We found that the 1PkN models with *k* < 13 could sufficiently describe the global fluctuation of DNA, whereas the 1P3N and 1P4N models are also applicable to detailed analyses of local fluctuations.

The 1PkN models can deal with sequence-dependent behavior at a more than 10 times reduced computational cost than required for the previous CG models. As a demonstration, we constructed 1P3N models of the entire *Plasmodium falciparum* and human mitochondrial genomes (5,967 and 16,569 bp, respectively), and analyzed their physical properties affecting local bending and twisting, which are important for the formation of nucleoid structures.

## Models and methods

### The 1P1N coarse-grained (CG) model

First, we briefly review our previous CG elastic network model of double-stranded DNA, where each nucleotide is represented by one particle, namely the 1P1N model (Isami et al., 2015).

Similar to other elastic network models, this model depends on a basic structure. To consider sequence-dependent geometry, we constructed a basic structure according to a set of helical parameters (base-step and base-pair parameters) depending on the local nucleotide sequences (typically two consecutive base-pairs). These parameters have been determined by experiments and molecular dynamics simulations (Olson et al., 1998, 2006; Lankaš et al., 2003; Perez et al., 2008; Morozov et al., 2009; Lavery et al., 2010; Dans et al., 2012; Hospital et al., 2013). Throughout this study, we employed the helical parameters obtained in *in vitro* experiments and from X-ray crystal structure analysis (Table S1) (Olson et al., 1998, 2006; Lankaš et al., 2003; Morozov et al., 2009; Freeman et al., 2014a), which were also used in other recent CG DNA models (Freeman et al., 2014a; Isami et al., 2015). Qualitatively similar results were obtained even if different helical parameter sets were applied (Perez et al., 2008; Lavery et al., 2010; Dans et al., 2012; Hospital et al., 2013) for all analyses described in this paper (data not shown). We used 3DNA (Lu and Olson, 2003) to obtain the coordinate of each atom in the DNA, on the basis of the helical parameters (for detailed methods on the generation of the atom coordinates, see Lu and Olson, 2003).

In the 1P1N model, **x**_*i*_ = (*x*_*i*_, *y*_*i*_, *z*_*i*_), the position of nucleotide *i* (*i* = 0, 1, 2, …), is given as the coordinate of the C1' carbon of that nucleotide, and *m*_*i*_, the mass of nucleotide *i*, is considered to be equal (*m*_*i*_ = *m*). The potential *V* of the interaction between nucleotides is given as

(1) $$\begin{array}{l} V=\underset{i,j}{\sum }\frac{C^{1}B_{ij}^{1}}{2}{\left(\left|{\text{x}}_{i}-{\text{x}}_{j}|-|{\text{x}}_{i}^{0}-{\text{x}}_{j}^{0}\right|\right)}^{2}, \end{array}$$

where ${\text{x}}_{i}^{0}$ is the position of nucleotide *i* in the basic structure. We assumed that nucleotides *i* and *i*^*c*^ form a base pair, i.e., the superscript ^*c*^ indicates the corresponding nucleotide in the complementary strand. $B_{ij}^{1}=1$ for *j* = *i* ± 2, *i* ± 1, (*i* ± 3)^*c*^, (*i* ± 2)^*c*^, (*i* ± 1)^*c*^, and *i*^*c*^; and *B*_*ij*_ = 0 otherwise. *C*^1^ is 7.7 kJ/(Å^2^ mol) (by rescaling $m=10^{-3}/N_{A}$ [kg]). Using normal-mode analysis (NMA), we confirmed that this model can accurately reproduce the dynamic parameters of each nucleotide obtained by means of the all-atom model, for the several types of helical parameters mentioned above (Isami et al., 2015).

### The 1PkN coarse-grained (CG) model

In this study, we aimed to construct further CG models intended for the analysis of long DNA. Here, we present 1PkN models, where *k* nucleotides are represented by a single particle. If we were to directly compare such models with the corresponding all-atom models, the computational cost of the analysis would be huge (e.g., a 450-bp model, considered below for the 1P9N case, involves 18,450 atoms), as we would need to assess a statistically sufficient number of sequences. Therefore, we used the 1P1N model as a reference to design and validate these new models.

**Figure 1:**
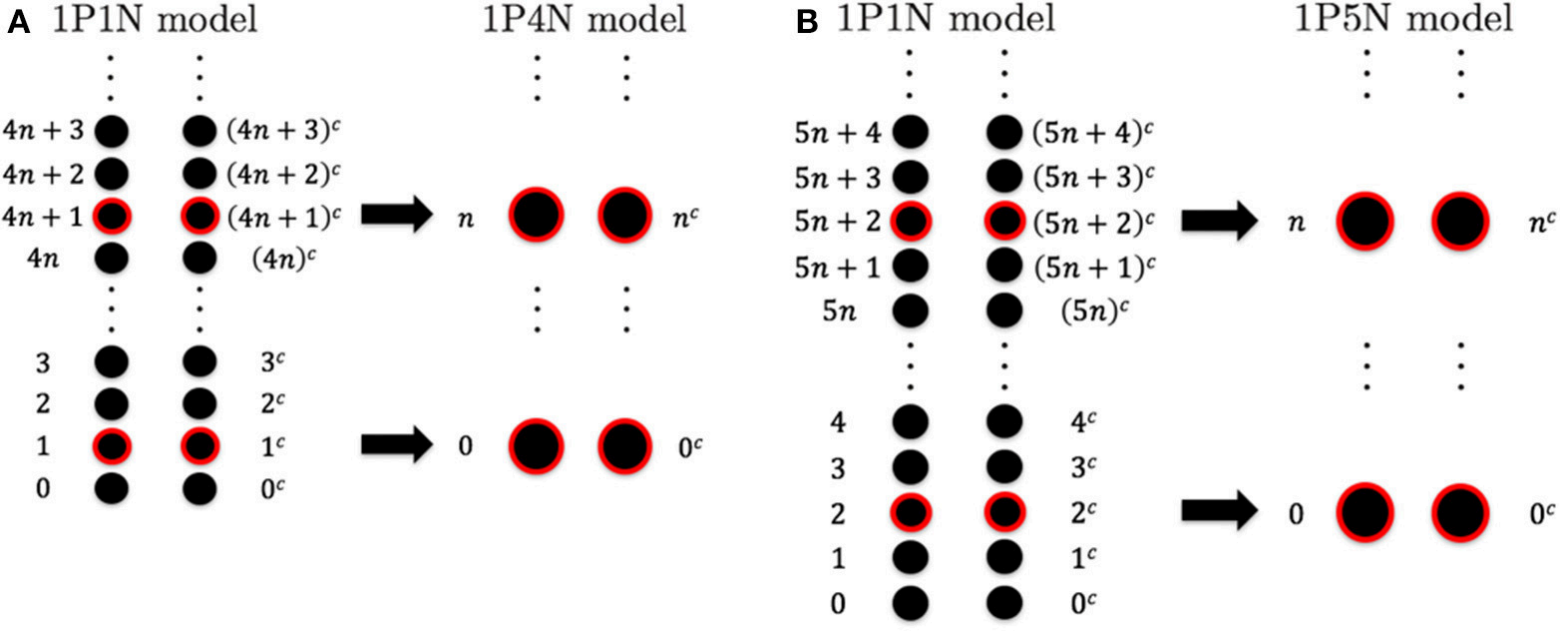
**Relationship between the particle indices of models 1P1N and 1PkN at (A)** *k* = 4 and **(B)** *k* = 5.

To construct the 1PkN model, each *k* particle (i.e., at the center of consecutive *k* bases) in the 1P1N model was extracted. Figure 1 shows the cases for *k* = 4 and *k* = 5. More specifically, we assumed a 1PkN model of *L* bp, consisting of $2\times \left[\frac{L}{k}\right]$ particles (where [ ] indicates the Gauss symbol). The three-dimensional coordinates **q**_*n*_, ${\text{q}}_{n^{c}}$ and mass *m*_*n*_, $m_{n^{c}}$ of the *n*-th (*n* = 0, 1, 2, ⋯) and *n*^*c*^-th particles in the 1PkN model were expressed as those of the *i*-th and *i*^*c*^-th particles (i.e., nucleotides) in the 1P1N model (i.e., **x**_*i*_, ${\text{x}}_{i^{c}}$, *m*_*i*_, and $m_{i^{c}}$), respectively, where *i* = *kn* + *k*_0_ and $i^{c}={\left(kn+k_{0}\right)}^{c}$ (the *n*-th and *n*^*c*^-th particles form a base pair). Here, $k_{0}=\frac{k-2}{2}$ (*k*: even) or $\frac{k-1}{2}$ (*k*: odd) (Figure 1).

**Figure 2:**
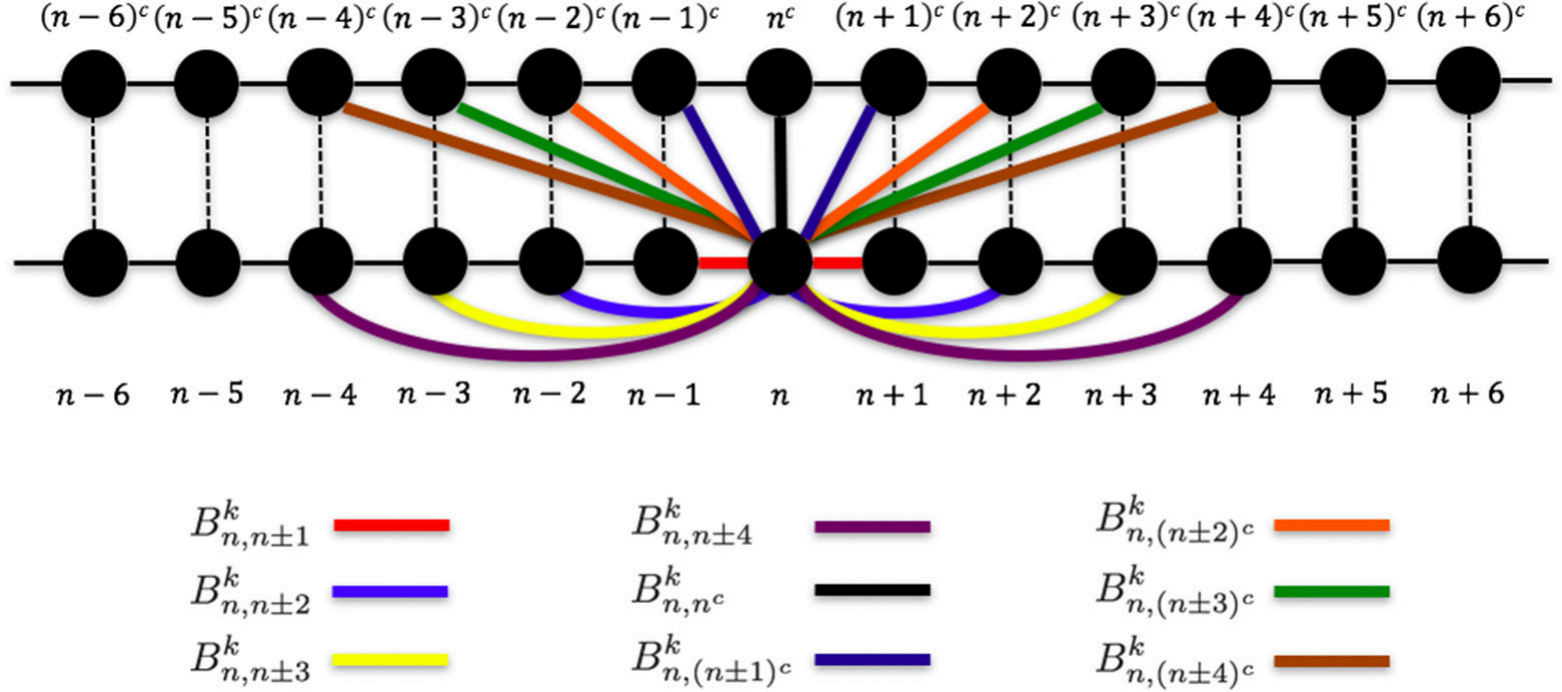
**Connections between particle *n* and other particles in the 1PkN model**.

Then, we define the interaction potential *V* between the particles as

(2) $$\begin{array}{l} V=\underset{n,l}{\sum }\frac{C^{k}B_{nl}^{k}}{2}{\left(\left|{\text{q}}_{n}-{\text{q}}_{l}|-|{\text{q}}_{n}^{0}-{\text{q}}_{l}^{0}\right|\right)}^{2}, \end{array}$$

where ${\text{q}}_{n}^{0}$ is the position of particle *n* in the basic structure, $B_{nl}^{k}$ indicates the weight of the connection between particles *n* and *l* (Figure 2), and *C*^*k*^ is the connection strength. We determined the appropriate *k*, *C*^*k*^, and $B_{nl}^{k}$ values as shown below.

As mentioned above, the basic structure was constructed according to a set of sequence-dependent helical parameters. Both the 1P1N model and 1PkN (*k* > 1) models incorporate sequence-dependent physical properties through the interaction potential *V* that depends on the basic structure (${\text{x}}_{i}^{0}$ or their subset ${\text{q}}_{n}^{0}$).

### Normal-mode analysis (NMA)

To optimize and evaluate the 1PkN model, we compared the structural fluctuations in the 1PkN model with those of the 1P1N model. For this purpose, NMA was used to estimate the mobility of each particle in the 1PkN model. NMA has been applied to elastic network models and is demonstrated to well reproduce the structural properties of biomolecules such as proteins (Tirion, 1996; Atilgan et al., 2001; Bahar and Rader, 2005; Yang et al., 2009; Bahar et al., 2009, 2010; Dykeman and Sankey, 2010). Here, we provide a brief overview of NMA.

We defined **q**(*t*) (**q** = (**q**_0_, **q**_1_, ⋯, **q**_*N*−1_), **q**_*n*_ = (*x*_*n*_, *y*_*n*_, *z*_*n*_)) as a 3*N*-dimensional position vector, and **q**^0^ as the position vector of the basic structure. Using linear approximation, small deviations δ**q**(*t*) = **q** − **q**^0^ around the basic structure obey the following equation:

(3) $$\begin{array}{l} \delta \text{q}\left(t\right)=\underset{w_{k}\neq 0}{\sum }A_{k}{\text{v}}^{k}e^{iw_{k}t}, \end{array}$$

where $-{\left(w_{k}\right)}^{2}$ and ${\text{v}}^{k}=\left({\text{v}}_{0}^{k},{\text{v}}_{1}^{k},\cdots \;,{\text{v}}_{N-1}^{k}\right)$ (${\text{v}}_{i}^{k}=\left(v_{x_{i}}^{k},v_{y_{i}}^{k},v_{z_{i}}^{k}\right)$, and $\underset{i}{\sum }|{\text{v}}_{i}^{k}{|}^{2}=1$) represent the *k*-th largest eigenvalue and its eigenvector of the 3*N* × 3*N* Hessian matrix **H**, where

(4) $$\begin{array}{r} \text{H}=\left(H_{nl}\right), \end{array}$$

(5) $$\begin{array}{r} H_{nl}=-{\left(\frac{\partial^{2}V}{\partial q_{n}\partial q_{l}}\right)}_{\text{q}={\text{q}}^{0}} \end{array}$$

We presumed that the system is in thermodynamic equilibrium at temperature *T*. Then, the amplitude *A*_*k*_ is expressed as

(6) $$\begin{array}{l} {\left(A_{k}\right)}^{2}=\frac{k_{B}T}{{\left(w_{k}\right)}^{2}m}, \end{array}$$

with the Boltzmann constant *k*_*B*_. Using this solution, the mean square fluctuation (MSF; i.e., the strength of thermal fluctuation) of the *n*-th particle and the correlation of motion between particles *n* and *l* were obtained as

(7) $$\begin{array}{l} F_{n}^{a}=<|\delta {\text{q}}_{n}{|}^{2}>=\underset{w_{k}\neq 0}{\sum }\frac{k_{B}T}{{\left(w_{k}\right)}^{2}m}|{\text{v}}_{n}^{k}{|}^{2} \end{array}$$

and

(8) $$\begin{array}{l} F_{nl}^{c}=\frac{<\delta {\text{q}}_{n}\cdot \delta {\text{q}}_{l}>}{\sqrt{F_{n}^{a}}\sqrt{F_{l}^{a}}}, \end{array}$$

respectively, where <…> represents the temporal average.

Similarly, for the 1P1N model, the MSF $G_{i}^{a}=<|\delta {\text{q}}_{i}{|}^{2}>$ and the correlation of motion $G_{ij}^{c}=<\delta {\text{q}}_{i}\cdot \delta {\text{q}}_{j}>/\left(\sqrt{G_{i}^{a}}\sqrt{G_{j}^{a}}\right)$ were calculated, where the *i*-th and *j*-th particles in the 1P1N model correspond to the *n*-th and *l*-th particles in the 1PkN model, respectively.

Note that the particles at the ends of the 1PkN model do not correspond to those at the ends of the 1P1N model. Furthermore, the behavior of the particles at the ends of these models often become specific because there are fewer interacting particles at the ends than in the middle. Therefore, in the estimation of fluctuations, we omitted δ**q**_*n*_ for four particles (i.e., two particle pairs) at each end.

### Determination of the parameters in the 1PkN model

The 1PkN model above includes the free parameters $B_{nl}^{k}$ and *C*^*k*^. We optimized these parameters to fit the overall fluctuation of each particle with that observed in the 1P1N model.

To compare the fluctuations between the 1P1N and 1PkN models, we employed randomly chosen DNA sequences with 50 × *k* base pairs for each *k*; i.e., the number of particle pairs in the 1PkN model was always 50. Pearson's correlation coefficients ρ^*a*^ between the MSF $F_{n}^{a}$ in the 1PkN and $G_{i}^{a}$ in the 1P1N models was used as the performance index. A set of $B_{nl}^{k}$ that maximizes the average of ρ^*a*^ for 500 randomly chosen sequences was determined by a systematic survey (Table 1). For the sake of simplicity, we assumed $B_{nl}^{k}$ for *l* = *n*±4, *n*±3, *n*±2, *n*±1, (*n*±4)^*c*^, (*n*±3)^*c*^, (*n*±2)^*c*^, (*n*±1)^*c*^, and *n*^*c*^ is equal to 0 or 1, and $B_{nl}^{k}=0$ otherwise (Figure 2). We confirmed that the results were qualitatively the same even when $B_{nl}^{k}$ could take intermediate values.

**Table 1:** **Set of appropriate *C*^*k*^ and $B_{nl}^{k}$ values for each *k* ∈ {1, 3, 4, 9, 13} showing the highest correlation of fluctuations between the 1PkN and 1P1N models**.

***k***	**1**	**3**	**4**	**9**	**13**
*C*^*k*^	7.7	7.2	0.7	0.2	0.1
$B_{n,\;n\;\pm \;1}^{k}$	1	1	1	1	1
$B_{n,\;n\;\pm \;2}^{k}$	1	1	1	1	1
$B_{n,\;n\;\pm \;3}^{k}$	0	0	0	1	1
$B_{n,\;n\;\pm \;4}^{k}$	0	0	0	0	0
$B_{n,\;n^{c}}^{k}$	1	1	1	1	1
$B_{n,\;{\left(n\;\pm \;1\right)}^{c}}^{k}$	1	1	1	1	1
$B_{n,\;{\left(n\;\pm \;2\right)}^{c}}^{k}$	1	0	1	1	1
$B_{n,\;{\left(n\;\pm \;3\right)}^{c}}^{k}$	1	0	0	1	1
$B_{n,\;{\left(n\;\pm \;4\right)}^{c}}^{k}$	0	0	0	0	1

*For other k values, see Table S2*.

*C*^*k*^ values for *k* > 1 were chosen manually, which alter only the absolute level of the fluctuations and do not affect the correlations we consider below.

### Comparison of anisotropic fluctuations in the 1P1N and 1PkN models

As previously demonstrated, the 1P1N model can effectively reproduce the fluctuations of an all-atom model in several typical geometric vector directions (Isami et al., 2015). To evaluate the performance of the 1PkN models, we again adopted anisotropic fluctuations as indices, and compared them between the 1P1N and 1PkN models for randomly chosen DNA sequences. Since the total numbers of modes are different between the 1P1N and 1PkN models for *k* > 2, we cannot compare each of their eigenvectors directly. Thus, we focused on anisotropic MSFs in several representative directions for each particle in the 1P1N and 1PkN models.

**Figure 3:**
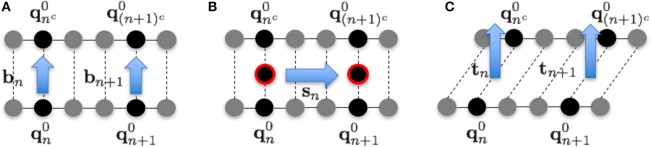
**Illustration of vectors in the (A)** base pair direction **b**_*n*_, **(B)** chain direction **s**_*n*_, and **(C)** surface vector direction **t**_*n*_, for *k* = 3.

The fluctuations in each direction were evaluated as follows. In the 1PkN model, the *n*-th and *n*^*c*^-th particles form a base pair. We defined the unit vectors

(9) $$\begin{array}{r} {\text{b}}_{n}=\frac{{\text{q}}_{n^{c}}^{0}-{\text{q}}_{n}^{0}}{\left|{\text{q}}_{n^{c}}^{0}-{\text{q}}_{n}^{0}\right|}, \end{array}$$

(10) $$\begin{array}{r} {\text{s}}_{n}=\frac{\left({\text{q}}_{n\;+\;1}^{0}\;+\;{\text{q}}_{{\left(n\;+\;1\right)}^{c}}^{0}\right)-\left({\text{q}}_{n}^{0}\;+\;{\text{q}}_{n^{c}}^{0}\right)}{\left|\left({\text{q}}_{n\;+\;1}^{0}\;+\;{\text{q}}_{{\left(n\;+\;1\right)}^{c}}^{0}\right)-\left({\text{q}}_{n}^{0}\;+\;{\text{q}}_{n^{c}}^{0}\right)\right|}, \end{array}$$

and

(11) $$\begin{array}{l} {\text{t}}_{n}=\frac{{\text{s}}_{n}\times {\text{b}}_{n}}{\left|{\text{s}}_{n}\times {\text{b}}_{n}\right|} \end{array}$$

to the directions shown in Figure 3. Although **b**_*n*_ and **s**_*n*_ are not orthogonal in general, we confirmed that the angles of these vectors were always sufficiently close to $\frac{\pi }{2}$ rad for each *n*.

Using these vectors, we decomposed the MSFs of the *n*-th and *n*^*c*^-th particles of the 1PkN model (i.e., $F_{n}^{a}$ and $F_{n^{c}}^{a}$) into these three directions to obtain $F_{n}^{b}$ ($F_{n^{c}}^{b}$), $F_{n}^{s}$ ($F_{n^{c}}^{s}$), and $F_{n}^{t}$ ($F_{n^{c}}^{t}$). We also calculated the MSF of relative particle positions $DF_{n}^{a}$ of the *n*-th particle pair, and its projection $DF_{n}^{b}$, $DF_{n}^{s}$, and $DF_{n}^{t}$ to these three directions. Furthermore, we estimated the local flexibility toward the directions of bending and twisting around the *n*-th particle pair, as denoted by $F_{n}^{\text{bend}}$ and $F_{n}^{\text{twist}}$, respectively. The definitions of these indices are provided in Table 2.

**Table 2:** **Indices of fluctuations in the 1PkN and 1P1N models**.

**Meaning**	**Definition**	**Correlation**
MSF (mean	$F_{n}^{a}=<|\delta {\text{q}}_{n}{|}^{2}>$, ($F_{n^{c}}^{a}=<|\delta {\text{q}}_{n^{c}}{|}^{2}>$)	ρ^*a*^
square fluctuation)	$G_{i}^{a}=<|\delta {\text{q}}_{i}{|}^{2}>$, ($G_{i^{c}}^{a}=<|\delta {\text{q}}_{i^{c}}{|}^{2}>$)	
MSF parallel to ***b***_*n*_	$F_{n}^{b}=<|\delta {\text{q}}_{n}\cdot {\text{b}}_{n}{|}^{2}>$, ($F_{n^{c}}^{b}=<|\delta {\text{q}}_{n^{c}}\cdot {\text{b}}_{n}{|}^{2}>$)	ρ^*b*^
	$G_{i}^{b}=<|\delta {\text{q}}_{i}\cdot {\text{b}}_{i}^{1}{|}^{2}>$, ($G_{i^{c}}^{b}=<|\delta {\text{q}}_{i^{c}}\cdot {\text{b}}_{i}^{1}{|}^{2}>$)	
MSF parallel to ***s***_*n*_	$F_{n}^{s}=<|\delta {\text{q}}_{n}\cdot {\text{s}}_{n}{|}^{2}>$, ($F_{n^{c}}^{s}=<|\delta {\text{q}}_{n^{c}}\cdot {\text{s}}_{n}{|}^{2}>$)	ρ^*s*^
	$G_{i}^{s}=<|\delta {\text{q}}_{i}\cdot {\text{s}}_{i}^{1}{|}^{2}>$, ($G_{i^{c}}^{s}=<|\delta {\text{q}}_{i^{c}}\cdot {\text{s}}_{i}^{1}{|}^{2}>$)	
MSF parallel to ***t***_*n*_	$F_{n}^{t}=<|\delta {\text{q}}_{n}\cdot {\text{t}}_{n}{|}^{2}>$, $F_{n^{c}}^{t}=<|\delta {\text{q}}_{n^{c}}\cdot {\text{t}}_{n}{|}^{2}>$	ρ^*t*^
	$G_{i}^{t}=<|\delta {\text{q}}_{i}\cdot {\text{t}}_{i}^{1}{|}^{2}>$, ($G_{i^{c}}^{t}=<|\delta {\text{q}}_{i^{c}}\cdot {\text{t}}_{i}^{1}{|}^{2}>$)	
Inter-strand MSF	$DF_{n}^{a}=<|\delta {\text{q}}_{n}-\delta {\text{q}}_{n^{c}}{|}^{2}>$	ρ^*Da*^
	$DG_{i}^{a}=<|\delta {\text{q}}_{i}-\delta {\text{q}}_{i^{c}}{|}^{2}>$	
Inter-strand MSF	$DF_{n}^{b}=<|\left(\delta {\text{q}}_{n}-\delta {\text{q}}_{n^{c}}\right)\cdot {\text{b}}_{n}{|}^{2}>$	ρ^*Db*^
parallel to ***b***_*n*_	$DG_{i}^{b}=<|\left(\delta {\text{q}}_{i}-\delta {\text{q}}_{i^{c}}\right)\cdot {\text{b}}_{i}^{1}{|}^{2}>$	
Inter-strand MSF	$DF_{n}^{s}=<|\left(\delta {\text{q}}_{n}-\delta {\text{q}}_{n^{c}}\right)\cdot {\text{s}}_{n}{|}^{2}>$	ρ^*Ds*^
parallel to ***s***_*n*_	$DG_{i}^{s}=<|\left(\delta {\text{q}}_{i}-\delta {\text{q}}_{i^{c}}\right)\cdot {\text{s}}_{i}^{1}{|}^{2}>$	
Inter-strand MSF	$DF_{n}^{t}=<|\left(\delta {\text{q}}_{n}-\delta {\text{q}}_{n^{c}}\right)\cdot {\text{t}}_{n}{|}^{2}>$	ρ^*Dt*^
parallel to ***t***_*n*_	$DG_{i}^{t}=<|\left(\delta {\text{q}}_{i}-\delta {\text{q}}_{i^{c}}\right)\cdot {\text{t}}_{i}^{1}{|}^{2}>$	
Local bending ability	$F_{n}^{\text{bend}}=<|\delta {\text{Q}}_{n+1}\cdot {\text{R}}_{n+1}-\delta {\text{Q}}_{n}\cdot {\text{R}}_{n}{|}^{2}>$	ρ^bend^
	$G_{i}^{\text{bend}}=<|\delta {\text{Q}}_{i+k}\cdot {\text{R}}_{i+k}^{1}-\delta {\text{Q}}_{i}\cdot {\text{R}}_{i}^{1}{|}^{2}>$	
Local twisting ability	$F_{n}^{\text{twist}}=<|\left(\delta {\text{q}}_{n+1}-\delta {\text{q}}_{{\left(n+1\right)}^{c}}\right)\cdot {\text{t}}_{n+1}-\left(\delta {\text{q}}_{n}-\delta {\text{q}}_{n^{c}}\right)\cdot {\text{t}}_{n}{|}^{2}>$	ρ^twist^
	$G_{i}^{\text{twist}}=<|\left(\delta {\text{q}}_{i+k}-\delta {\text{q}}_{{\left(i+k\right)}^{c}}\right)\cdot {\text{t}}_{i+k}^{1}-\left(\delta {\text{q}}_{i}-\delta {\text{q}}_{i^{c}}\right)\cdot {\text{t}}_{i}^{1}{|}^{2}>$	
Correlation of motion	$F_{nl}^{c}=<\delta {\text{q}}_{n}\cdot \delta {\text{q}}_{l}>/\left(\sqrt{F_{n}^{a}}\sqrt{F_{l}^{a}}\right)$	ρ^*c*^
	$G_{ij}^{c}=<\delta {\text{q}}_{i}\cdot \delta {\text{q}}_{j}>/\left(\sqrt{G_{i}^{a}}\sqrt{G_{j}^{a}}\right)$	

*We defined* $\delta {\text{Q}}_{n}\;=\;\left(\frac{\delta {\text{q}}_{n}\;+\;\delta {\text{q}}_{n^{c}}}{2}\right)$, $\delta {\text{Q}}_{i}\;=\;\left(\frac{\delta {\text{q}}_{i}\;+\;\delta {\text{q}}_{i^{c}}}{2}\right)$, ***R**_n_ = (**b**_n_ + **t**_n_)*, ${\text{R}}_{i}^{1}\;=\;\left({\text{b}}_{i}^{1}\;+\;{\text{t}}_{i}^{1}\right)$.

Moreover, to compare the behavior between the *n*-th (*n*^*c*^-th) particle in the 1PkN model and the corresponding *i*-th (*i*^*c*^-th) particle in the 1P1N model, their motions must be projected onto the common vectors of the 1PkN model. For this purpose, we adopted ${\text{b}}_{i}^{1}={\text{b}}_{n}$, ${\text{s}}_{i}^{1}={\text{s}}_{n}$, and ${\text{t}}_{i}^{1}={\text{t}}_{n}$ for *i* = *kn*+*k*_0_ (**b**_*n*_, **s**_*n*_, and **t**_*n*_ were obtained from the 1PkN model). Using these vectors, we projected the MSFs of the *i*-th (*i*^*c*^-th) particles in the 1P1N model (i.e., $G_{i}^{a}$ ($G_{i^{c}}^{a}$)) onto the same directions as those of the corresponding *n*-th (*n*^*c*^-th) particles in the 1PkN model, to obtain $G_{i}^{b}$ ($G_{i^{c}}^{b}$), $G_{i}^{s}$ ($G_{i^{c}}^{s}$), and $G_{i}^{t}$ ($G_{i^{c}}^{t}$). Similarly, the MSFs of the relative particle positions of the *i*-th particle pair ($DG_{i}^{a}$, $DG_{i}^{b}$, $DG_{i}^{s}$, and $DG_{i}^{t}$) and the local flexibility values ($G_{i}^{\text{bend}}$ and $G_{i}^{\text{twist}}$) in the same directions were obtained (Table 2).

Similarities between the fluctuations in the 1PkN model ($F_{n}^{•}$ and $DF_{n}^{•}$) and those in the 1P1N model ($G_{i}^{•}$ and $DG_{i}^{•}$) for each sequence were evaluated by means of the Pearson's correlation coefficients ρ^•^ listed in Table 2. The correlations of motion, $F_{nl}^{c}$ and $G_{ij}^{c}$, were also compared using Pearson's correlation coefficient ρ^*c*^.

To compare the absolute magnitude of fluctuations, we define ${\langle F_{n}^{•}\rangle }_{n}$, ${\langle DF_{n}^{•}\rangle }_{n}$, ${\langle G_{i}^{•}\rangle }_{i}$, and ${\langle DG_{i}^{•}\rangle }_{i}$ as the average of $F_{n}^{•}$, $DF_{n}^{•}$, $G_{i}^{•}$, and $DG_{i}^{•}$, respectively, over the entire sequence except for two particle pairs (2 × *k* base pairs) at each end in the 1PkN (1P1N) model.

### Models of the mitochondrial genome

Using the 1P3N model, we analyzed the dynamics of the mitochondrial DNA of *P. falciparum* (5,967 bp, the shortest genome of all known mitochondrial DNA; NCBI Reference Sequence: NC_002375.1), human (16,569 bp; NCBI Reference Sequence: NC_012920.1), *Schizosaccharomyces pombe* (19,431 bp; NCBI Reference Sequence: NC_001326.1), and *Drosophila melanogaster* (19,524 bp; GenBank accession No.: KJ947872.2), using NMA. As the 3018-th base of human mitochondrial DNA remains unknown (http://hgdownload.soe.ucsc.edu/goldenPath/hg38/chromosomes/), we tried all four possible cases with adenine, guanine, cytosine, or thymine at that position.

We assessed the local bending and twisting ability $F_{n}^{\text{bend}}$ and $F_{n}^{\text{twist}}$, respectively, of the *n*-th particle pair (*i* = 3*n* + 1-th base pair). The moving average was taken over seven particle pairs: $MF_{n}^{\text{bend}}=\overset{n+6}{\underset{m=n}{\sum }}F_{m}^{\text{bend}}$ and $MF_{n}^{\text{twist}}=\overset{n+6}{\underset{m=n}{\sum }}F_{m}^{\text{twist}}$. The averaging window (21 bp) largely corresponds to the length of the histone-like protein TFAM-binding sequence (22 bp Alam et al., 2003; Pohjoismäki et al., 2006; Kaufman et al., 2007; Ngo et al., 2014).

## Results and discussion

### Validation of the 1PkN model by means of anisotropic fluctuations

**Figure 4:**
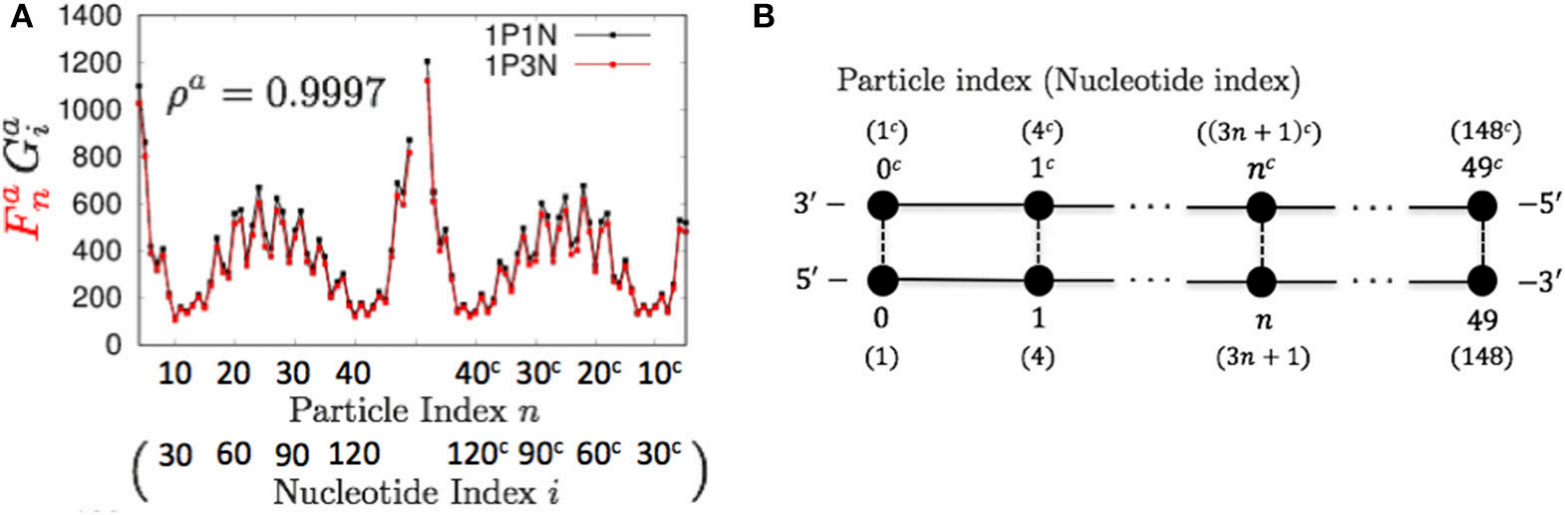
**Comparisons of the flctuations of particles between the 1P3N and 1P1N models for a typical 150-bp random sequence; (A)** $F_{n}^{a}$ and $G_{i}^{a}$. Black curves indicate the fluctuation profiles of the 1P1N model, and red curves show the fluctuation profiles of the 1P3N model. **(B)** Particle (nucleotide) indices.

**Figure 5:**
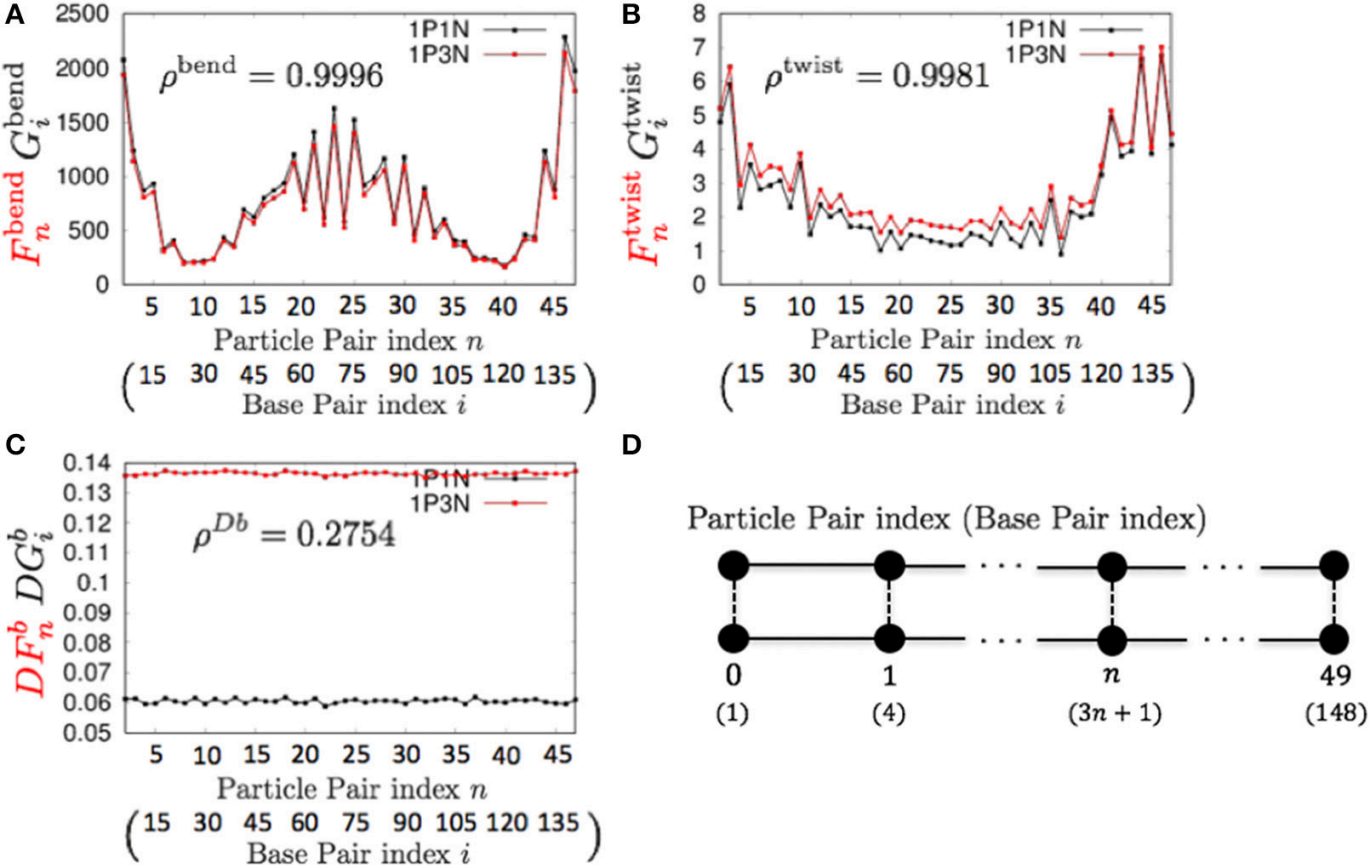
**Comparisons of the fluctuations of particle pairs between the 1P3N and 1P1N models for a typical 150-bp random sequence for (A)** $F_{n}^{\text{bend}}$ and $G_{i}^{\text{bend}}$, **(B)** $F_{n}^{\text{twist}}$ and $G_{i}^{\text{twist}}$, and **(C)** $DF_{n}^{b}$ and $DG_{i}^{b}$. Black curves indicate the fluctuation profiles of the 1P1N model, and red curves show the fluctuation profiles of the 1P3N model. **(D)** Particle pair (base pair) indices.

**Figure 6:**
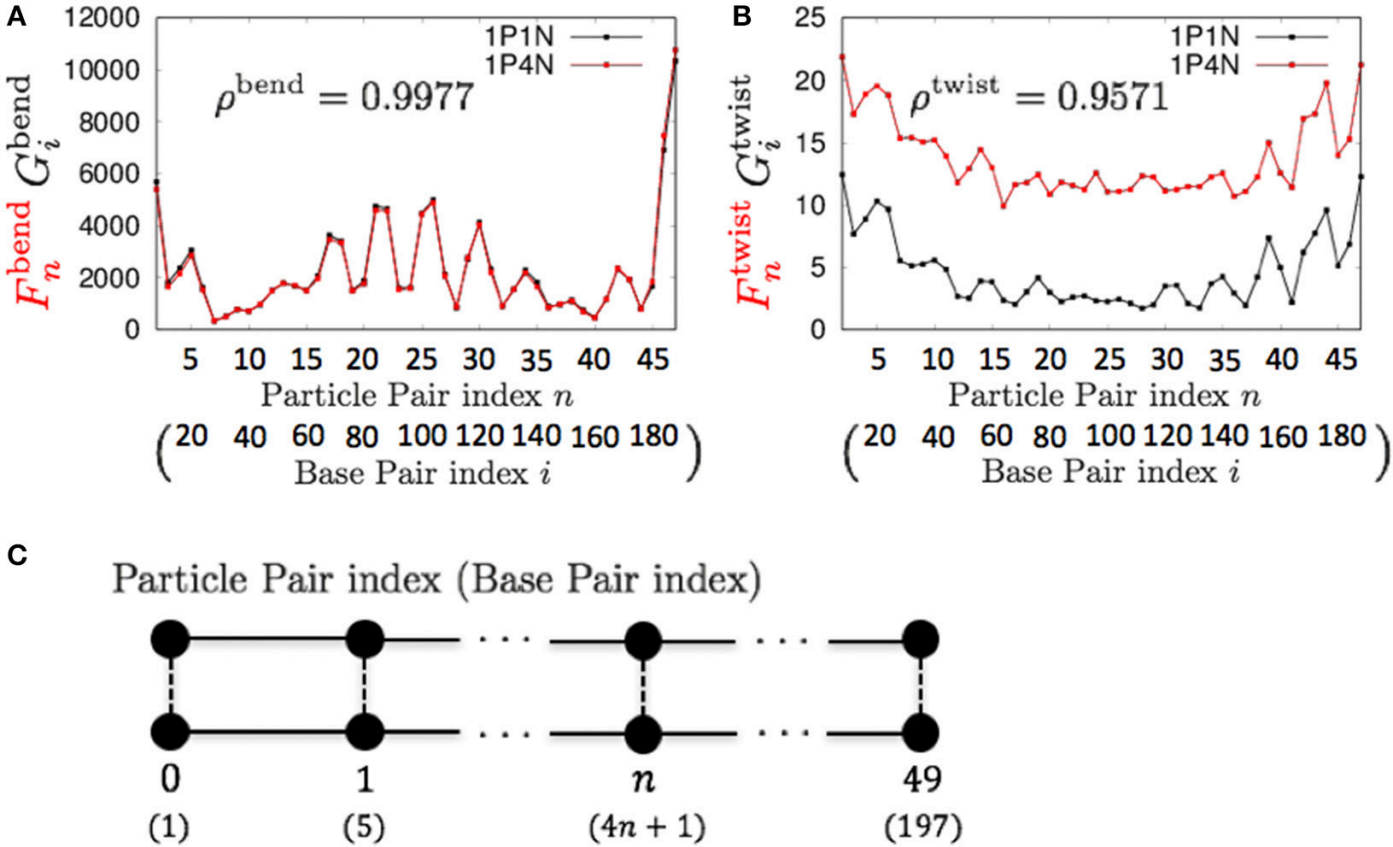
**Comparisons of the fluctuations of particles between the 1P4N and 1P1N models for a typical 200-bp random sequence for (A)** $F_{n}^{\text{bend}}$ and $G_{i}^{\text{bend}}$ and **(B)** $F_{n}^{\text{twist}}$ and $G_{i}^{\text{twist}}$. Black curves show the fluctuation profiles of the 1P1N model, and red curves show those of the 1P4N model. **(C)** Particle pair (base pair) indices.

**Figure 7:**
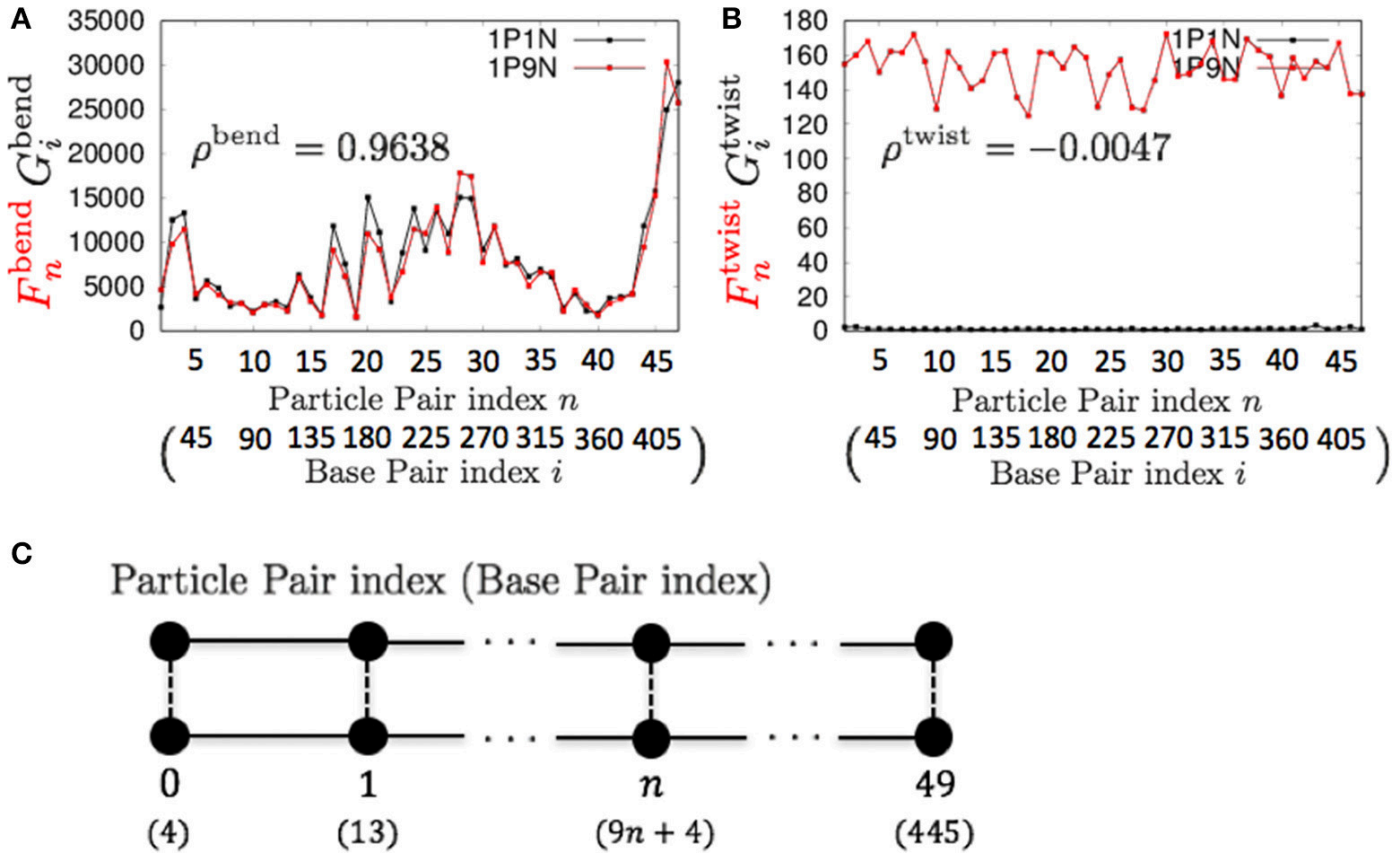
**Comparisons of the fluctuations of particles between the 1P9N and 1P1N models for a typical 450-bp random sequence for (A)** $F_{n}^{\text{bend}}$ and $G_{i}^{\text{bend}}$, and **(B)** $F_{n}^{\text{twist}}$ and $G_{i}^{\text{twist}}$. Black curves indicate the fluctuation profiles of the 1P1N model, and red curves indicate the fluctuation profiles of the 1P9N model. **(C)** Particle pair (base pair) indices.

As described in the previous section, we evaluated the 1PkN model using the indices listed in Table 2. We calculated these indices for each DNA sequence. For most *k* values up to *k* = 15, the profiles of MSFs of the *n*-th particle ($F_{n}^{a}$, $F_{n}^{b}$, $F_{n}^{s}$, and $F_{n}^{t}$) and the correlations of fluctuations between the *n*-th and *l*-th particles ($F_{nl}^{c}$) in the 1PkN model were similar to the corresponding values ($G_{i}^{a}$, $G_{i}^{b}$, $G_{i}^{s}$, $G_{i}^{t}$, and $G_{ij}^{c}$) obtained in the 1P1N model (Figures 4–**7**; Figures S1–S16). In particular, good agreement was observed between $F_{n}^{a}$ and $G_{i}^{a}$ (the overall fluctuation profiles are independent of the value of *C*^*k*^ because *C*^*k*^ influences only the absolute values of fluctuations). For the 500 randomly chosen sequences, ρ^*a*^, ρ^*b*^, ρ^*s*^, ρ^*t*^, and ρ^*c*^ showed high average values with low standard deviations for all *k* values considered (Table 3; details of ρ^•^ values and the profiles of $G_{i}^{•}-F_{n}^{•}$ ($DG_{i}^{•}-DF_{n}^{•}$) are shown in Tables S3–S5, Figures S1–S16).

**Table 3:** **Average (Avg.) and standard deviation (S.D.) values of the correlations between the fluctuations of the 1PkN and 1P1N models for 500 randomly chosen 50 × *k* bp sequences for *k* ∈ {3, 4, 9, 13}**.

***k* (bp)**	**Fluctuation**	**3 (150 bp)**	**4 (200 bp)**	**9 (450 bp)**	**13 (650 bp)**	**3 (450 bp)**
Avg. of ρ^*a*^	$F_{n}^{a}-G_{i}^{a}$	0.9993	0.9967	0.9511	0.8879	0.9998
(S.D. of ρ^*a*^)		(0.0006)	(0.0014)	(0.0320)	(0.0768)	(0.0002)
Avg. of ρ^*c*^	$F_{n}^{c}-G_{i}^{c}$	0.9998	0.9991	0.9870	0.9712	0.9999
(S.D. of ρ^*c*^)		(0.0001)	(0.0002)	(0.0094)	(0.0207)	(0.0001)
Avg. of ρ^bend^	$F_{n}^{\text{bend}}-G_{i}^{\text{bend}}$	0.9992	0.9980	0.9451	0.8754	0.9998
(S.D. of ρ^bend^)		(0.0005)	(0.0010)	(0.0474)	(0.0946)	(0.0001)
Avg. of ρ^twist^	$F_{n}^{\text{twist}}-G_{i}^{\text{twist}}$	0.9975	0.9549	−0.0028	−0.2917	0.9992
(S.D. of ρ^twist^)		(0.0014)	(0.0163)	(0.1566)	(0.1578)	(0.0005)
Avg. of ρ^*Db*^	$F_{n}^{Db}-G_{i}^{Db}$	0.2789	0.1908	−0.0695	0.0187	0.2769
(S.D. of ρ^*Db*^)		(0.1210)	(0.1526)	(0.1514)	(0.1458)	(0.0691)

*In addition, in the case of k = 3, randomly chosen 450-bp sequences are also inserted*.

For all *k* values considered, the absolute values of ρ^*Db*^ were always much lower (Table 3, Tables S3–S5). In fact, the amplitude of $DF_{n}^{b}$ was shown to be negligible, i.e., much smaller than those of the fluctuations in other directions (Figures S2, S6, S10, S14). From these results, $DF_{n}^{s}$ and $DF_{n}^{t}$ were presumed to be essential for characterization of the inter-strand motions of double-stranded DNA. Focusing directly on the local bending and twisting flexibility of DNA in detail, $F_{n}^{\text{bend}}$ and $F_{n}^{\text{twist}}$ were thought to be even more important than $DF_{n}^{s}$ and $DF_{n}^{t}$. Therefore, we mainly focused on ρ^bend^ and ρ^twist^ for the comparison between the 1PkN and 1P1N models.

For *k* = 3, ρ^bend^ and ρ^twist^ showed particularly high values (Table 3 and Figure 5). Therefore, the 1P3N model with an appropriate $B_{nl}^{k}$ can effectively reproduce the mechanical properties, including local bending and twisting ability, of the 1P1N model; thus, although indirectly, the 1P3N model was proven to well reproduce such properties of the all-atom model. In addition, we confirmed that the result was almost identical for longer (450 bp) sequences (Table 3, Figures S13–S16), suggesting that the high correlations between the 1P3N and 1P1N models were independent of the sequence length.

For *k* = 4, ρ^bend^ and ρ^twist^ were sufficiently high (greater than 0.95), although a non-negligible difference exists between the absolute values of $F_{n}^{\text{twist}}$ and $G_{i}^{\text{twist}}$ (Figure 6). Therefore, if we analyze only the relative patterns of the local bending and twisting flexibility of DNA, the 1P4N model seems to be sufficiently useful.

The local twisting flexibility was not well reproduced for *k* > 4. This reflects the fact that the pitch of B-DNA is 10–11 base pairs, and hence the right-handed double-stranded structure was not retained for *k* = 5 (i.e., 2 particle pairs per pitch) or larger.

These 1P3N and 1P4N models contain 1/3 and 1/4 of the particles, respectively, as compared to the 1P1N model. Although the computational cost of the NMA of these models is much lower (by a factor of 1/10 to 1/30 for *k* = 3) than that of the 1P1N model, the accuracy of the obtained statistical results is almost identical (the computational cost to obtain all the eigenvalues and eigenvectors of an *N* × *N* matrix is generally between *O*(*N*^2^) and *O*(*N*^3^), which is the most time-consuming step; hence the reduction is expected to be *k*^−2^ to *k*^−3^).

Therefore, these models can be used for exhaustive analyses of the dynamic features of large DNA molecules. It should also be noted that ρ^*a*^, ρ^*b*^, ρ^*s*^, ρ^*t*^, ρ^bend^, and ρ^*c*^ were higher than 0.9 for all *k* < 13, suggesting that 1PkN models with larger *k* values (e.g., 1P9N in Figure 7) may be useful for analyses of only the crude sequence-dependent behavior of very long DNA molecules.

### Effects of the GC content on the magnitude of fluctuations

Next, to confirm the validity of our 1PkN model, we computed the magnitude of fluctuations in the 1P3N model, ${\langle F_{n}^{•}\rangle }_{n}$ and ${\langle DF_{n}^{•}\rangle }_{n}$, and in the 1P1N model, ${\langle G_{i}^{•}\rangle }_{i}$ and ${\langle DG_{i}^{•}\rangle }_{i}$, as representative cases. Here, we used 1000 randomly generated 150-bp sequences, each satisfying the designated GC-content value (rate of GC base pairs in the sequence) 0, 0.1, 0.2, …, 1.

**Figure 8:**
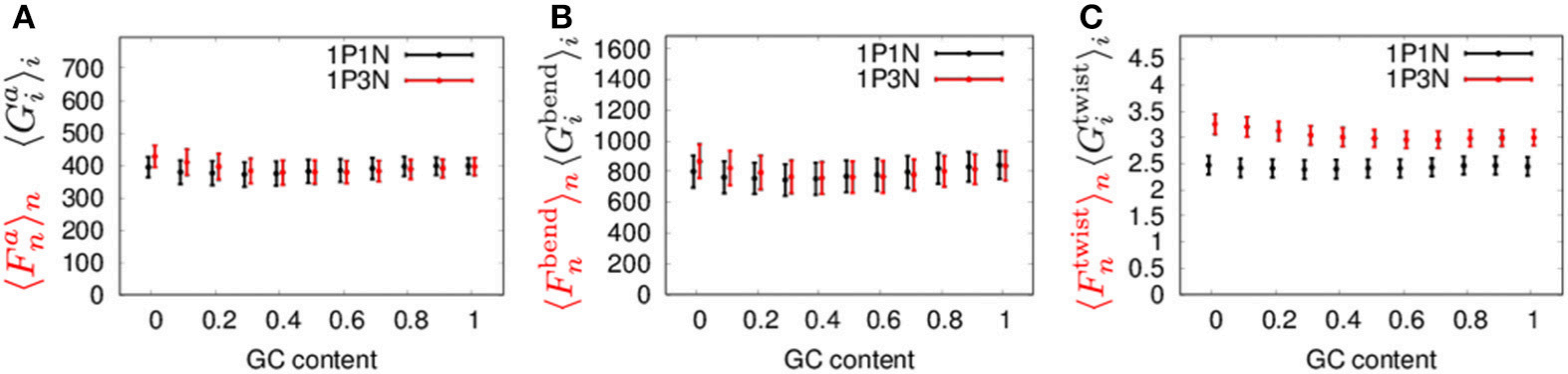
**Average ± standard deviations of (A)** ${\langle F_{n}^{a}\rangle }_{n}$ and ${\langle G_{i}^{a}\rangle }_{i}$, **(B)** ${\langle F_{n}^{\text{bend}}\rangle }_{n}$ and ${\langle G_{i}^{\text{bend}}\rangle }_{i}$, and **(C)** ${\langle F_{n}^{\text{twist}}\rangle }_{n}$ and ${\langle G_{i}^{\text{twist}}\rangle }_{i}$ for 1,000 samples of random 150-bp sequences with an average GC-content = 0, 0.1, 0.2, ⋯ 1.

The MSF exhibited approximately the same magnitude in the 1P3N model and 1P1N model for a GC-content of around 0.5, although in the case of lower GC-contents, ${\langle F_{n}^{a}\rangle }_{n}$ of the 1P3N model was slightly larger than ${\langle G_{i}^{a}\rangle }_{i}$ of the 1P1N model (specifically, reaching up to 10% larger) (Figure 8). Most of the profiles of ${\langle F_{n}^{•}\rangle }_{n}$ and ${\langle DF_{n}^{•}\rangle }_{n}$ were close to those of ${\langle G_{i}^{•}\rangle }_{i}$ and ${\langle DG_{i}^{•}\rangle }_{i}$; however, some cases resulted in considerably different magnitude profiles (Figure 8, Figure S17). Nevertheless, the 1P3N model consistently showed good agreement with the behavior of the 1P1N model.

### Application example: mitochondrial-genome dynamics

As an example of the possible application of the 1P3N model, we analyzed the whole-genome dynamics of mitochondrial DNA. Using the NMA of the 1P3N model constructed for these sequences, the potential for bending and superhelix formation in each local part of these genomes was inferred.

**Figure 9:**
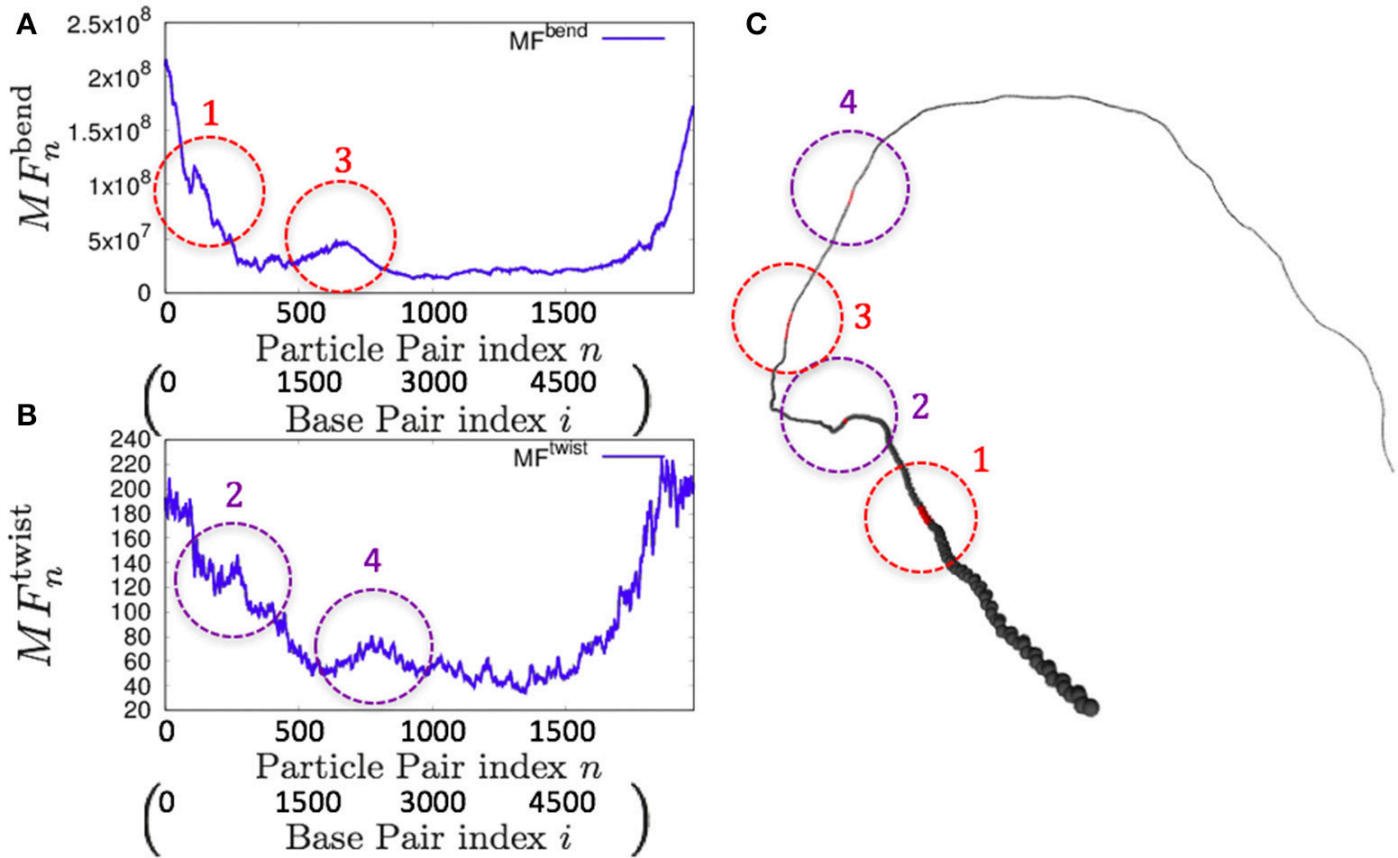
**Fluctuations and basic structure of *Plasmodium falciparum* mitochondrial DNA. (A)** Distribution of bending fluctuations. **(B)** Distribution of twisting fluctuations. **(C)** Basic structure of the analyzed genome and the corresponding regions analyzed in **(A,B)**.

**Figure 10:**
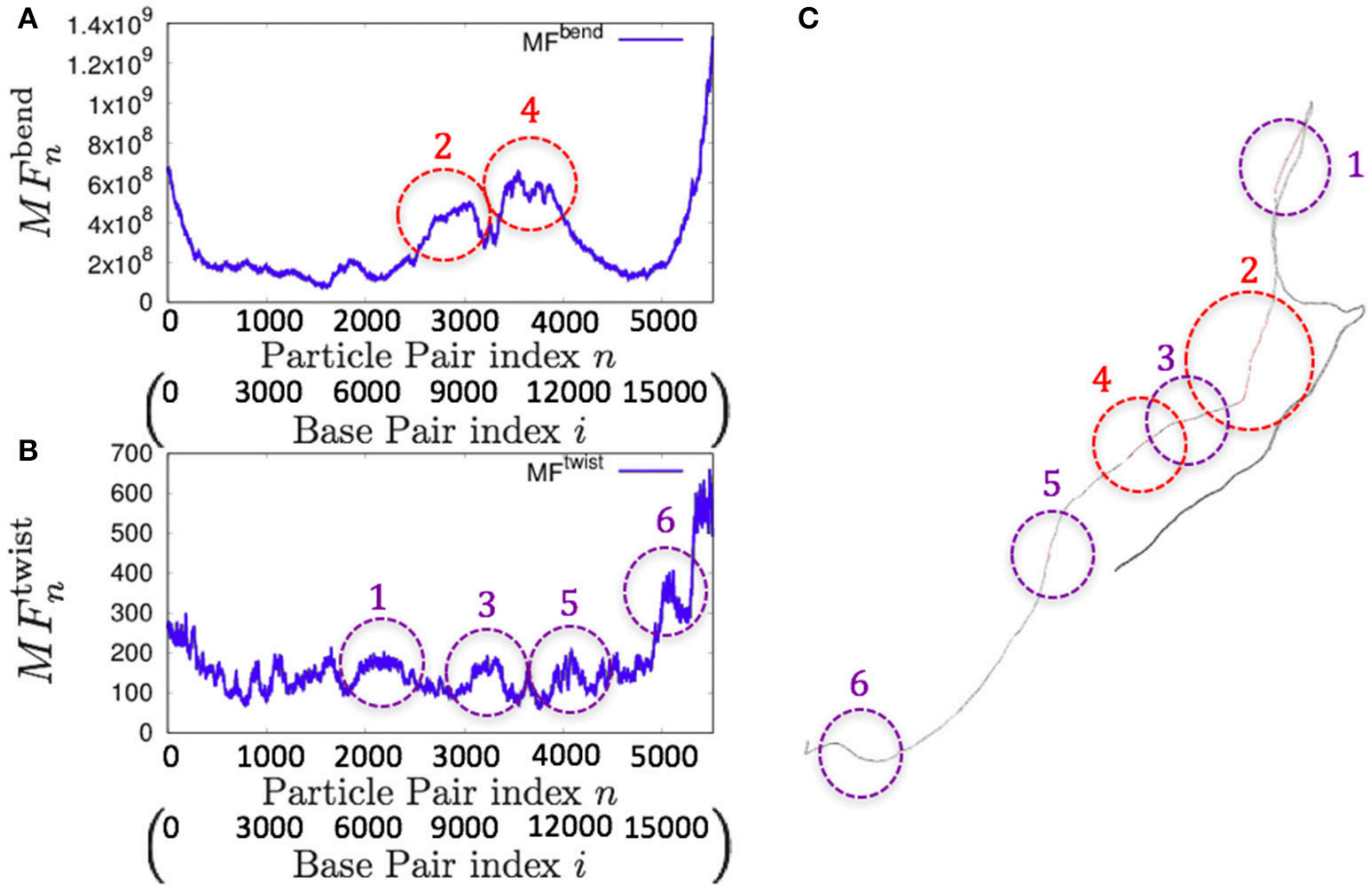
**Fluctuations of the *n*-th particle pair (*i*-th base pair) and the basic structure of human mitochondrial DNA in the case of an unknown base assumed to be adenine. (A)** Distribution of bending fluctuations. **(B)** Distribution of twisting fluctuations. **(C)** Basic structure of the analyzed genome and the corresponding regions analyzed in **(A,B)**.

Consistently, there were non-uniform profiles of $MF_{n}^{\text{bend}}$ and $MF_{n}^{\text{twist}}$ detected in *P. falciparum* mitochondrial DNA (Figure 9) and human mitochondrial DNA (Figure 10 and Figures S18–S20). Similar results were obtained for *S. pombe* (19,431 bp) and *D. melanogaster* (19,524 bp) mitochondrial DNA molecules, which are longer than human mitochondrial DNA (Figures S21, S22).

## Summary and conclusions

In this study, we developed the 1PkN model, which is more coarse-grained than recent DNA models and is intended for the analysis of the dynamic properties of long DNA molecules. In particular, we showed that the 1P3N model, where each particle represents three nucleotides, could accurately reproduce the distributions of the anisotropic fluctuations of nucleotides and the flexibility of local regions observed in the 1P1N model. Considering our previous result showing that the 1P1N model well reproduced the fluctuations of all-atom models of DNA (Isami et al., 2015), although indirectly, the 1P3N model was here shown to be in good agreement with all-atom models.

The 1P3N model seems to be useful for detailed analyses of the mechanical properties of long DNA molecules. The 1P4N model appears to be helpful for assessment of the fluctuations and flexibility of local regions as well. Using the 1P3N model, we analyzed the physical properties of whole mitochondrial genomes, even longer than 10^4^ bp. From the macroscopic perspective, our models should be connected to further coarse-grained polymer-like models (e.g., Brackley et al., 2013), where the sequence-dependent properties that emerged in our model could be incorporated as the inhomogeneity of mechanical parameters such as persistence length. Such multiscale approaches would be beneficial for analyses of huge structures such as whole bacterial genome DNA.

In this study, our analysis depended on the NMA. Moreover, we can perform molecular dynamics simulations of the 1P3N model, as well as the other proposed models, for the analysis of large deformations and interactions with a variety of proteins. These simulations would be useful to elucidate the formation and dynamics of compact nucleoid structures and ~30-nm chromatin structures (Gall, 1966; Adolph, 1980; Langmore and Paulson, 1983; Paulson and Langmore, 1983; Eltsov et al., 2008; Joti et al., 2012) or even larger structures such as the topologically associated domains in the nucleus (Zhu et al., 2007; Grosberg, 2012). Characteristics of DNA also depend on the conditions of the solution, such as temperature and ionic strength (Hamelberg et al., 2000; Mantz et al., 2007; Middleton et al., 2007). Therefore, adjustment of the model to reflect different conditions of solution would be warranted. We are planning to modify the proposed CG model to include the chemical properties of DNA and to analyze chromatin structures using not only NMA but also molecular dynamics simulations.

## Author contributions

Conceived and designed the experiments: AA and NS. Performed the experiments: TK and SI. Analyzed the data: TK, SI, YT, and AA. Wrote the paper: TK, YT, HN, NS, and AA.

## Funding

This work was partially supported by the Platform Project for Support of Drug Discovery and Life Science Research (Platform for Dynamic Approaches to a Living System) from the Ministry of Education, Culture, Sports, Science and Technology of Japan (MEXT), and the Japan Agency for Medical Research and Development (AMED); and by the Grant-in-Aid for Scientific Research on Innovative Areas, Initiative for High-Dimensional Data-Driven Science through Deepening of Sparse Modeling [grant numbers 4503 and 26120525] of the MEXT. Funding for the open-access fee: AMED.

### Conflict of interest statement

The authors declare that the research was conducted in the absence of any commercial or financial relationships that could be construed as a potential conflict of interest.
