# Supplementary material for: The 1-Particle-per-k-Nucleotides (1PkN) Elastic Network Model of DNA Dynamics with Sequence-Dependent Geometry
Source: Front Physiol. 2017 Mar 14;8:103. doi: 10.3389/fphys.2017.00103 (PMC5361685; doi:10.3389/fphys.2017.00103)
Supplement: Supplementary file 1 [file Presentation1.pdf]

Supplementary Material:
The 1-Particle-per-k-Nucleotides (1PkN) Elastic Network Model of DNA Dynamics with Sequence-Dependent Geometry

Takeru Kameda, Shuhei Isami, Yuichi Togashi *, Hiraku Nishimori, Naoaki Sakamoto and Akinori Awazu *

*Correspondence:
Akinori Awazu, Yuichi Togashi
awa@hiroshima-u.ac.jp, togashi@hiroshima-u.ac.jp

SUPPLEMENTARY DATA

Table S1

The helical parameter set obtained by *in vitro* experiments and X-ray crystal structure analysis (Freeman et al. (2014a); Olson et al. (1998, 2006); Morozov et al. (2009)).

Base-step parameters						
	Shift [Å]	Slide [Å]	Rise [Å]	Tilt [°]	Roll [°]	Twist [°]
AA	−0.05	−0.21	3.27	−1.84	0.76	35.31
AT	0.00	−0.56	3.39	0.00	−1.39	31.21
AC	0.21	−0.54	3.39	−0.64	−1.39	31.52
AG	0.12	−0.27	3.38	−1.48	3.15	33.05
TA	0.00	0.03	3.34	0.00	5.25	36.20
TT	0.05	−0.21	3.27	1.84	0.76	35.31
TC	0.27	−0.03	3.35	1.52	3.87	34.80
TG	0.16	0.18	3.38	0.05	5.95	35.02
CA	−0.27	−0.03	3.35	−1.52	3.87	34.80
CT	−0.12	−0.27	3.38	1.48	3.15	33.05
CC	0.02	−0.47	3.28	0.40	3.86	33.17
CG	0.00	0.57	3.49	0.00	4.29	35.30
GA	−0.27	−0.03	3.35	−1.52	3.87	34.80
GT	−0.21	−0.54	3.39	0.64	0.91	31.52
GC	0.00	−0.07	3.38	0.00	0.67	34.38
GG	−0.02	−0.47	3.28	−0.40	3.86	33.17
Base pair parameters						
	Shear [Å]	Stretch [Å]	Stagger [Å]	Buckle [°]	Propeller [°]	Opening [°]
A-T	0.07	−0.19	0.07	1.80	−15.00	1.50
T-A	−0.07	−0.19	0.07	−1.80	−15.00	1.50
C-G	0.16	−0.17	0.15	−4.90	−8.70	−0.60
G-C	−0.16	−0.17	0.15	4.90	−8.70	−0.60

Table S2

Set of appropriate C^k and B^k_{nl} values for each $k = 1 \cdots 15$ showing the highest correlation of fluctuations between the 1PkN and 1P1N models.

k	1	2	3	4	5	6	7	8	9	10	11	12	13	14	15
C^k	7.7	2.9	7.2	0.7	0.4	0.4	1.8	4.0	0.2	0.8	3.3	3.5	0.1	1.3	0.25
$B^k_{n,n\pm1}$	1	1	1	1	1	1	1	1	1	1	1	1	1	1	1
$B^k_{n,n\pm2}$	1	1	1	1	1	1	1	1	1	1	1	1	1	1	1
$B^k_{n,n\pm3}$	0	1	0	0	1	0	0	0	1	1	1	1	1	0	0
$B^k_{n,n\pm4}$	0	0	0	0	0	0	0	0	0	0	0	0	0	0	0
B^k_{n,n^c}	1	1	1	1	1	1	1	1	1	1	1	1	1	1	1
$B^k_{n,(n\pm1)^c}$	1	1	1	1	1	1	1	1	1	1	1	1	1	1	1
$B^k_{n,(n\pm2)^c}$	1	1	0	1	1	1	0	0	1	1	1	0	1	0	1
$B^k_{n,(n\pm3)^c}$	1	0	0	0	0	0	0	0	1	1	0	0	1	0	0
$B^k_{n,(n\pm4)^c}$	0	0	0	0	0	0	0	0	0	0	0	0	1	0	0

Table S3

Average (Avg.) and standard deviation (S.D.) values of the correlations between the fluctuations of the 1PkN and 1P1N models for 500 randomly chosen $50 \times k$ bp sequences for $k = 2 \cdots 8$.

k	2	3	4	5	6	7	8
Avg. of ρ^a (S.D. of ρ^a)	0.9947 (0.0007)	0.9993 (0.0006)	0.9967 (0.0014)	0.9931 (0.0041)	0.9905 (0.0066)	0.9946 (0.0039)	0.9897 (0.0162)
Avg. of ρ^b (S.D. of ρ^b)	0.9949 (0.0007)	0.9992 (0.0005)	0.9973 (0.0013)	0.9921 (0.0051)	0.9908 (0.0084)	0.9940 (0.0048)	0.9870 (0.0184)
Avg. of ρ^s (S.D. of ρ^s)	0.9955 (0.0009)	0.9991 (0.0005)	0.9970 (0.0014)	0.9930 (0.0051)	0.9915 (0.0070)	0.9935 (0.0053)	0.9936 (0.0105)
Avg. of ρ^t (S.D. of ρ^t)	0.9951 (0.0008)	0.9991 (0.0005)	0.9961 (0.0018)	0.9914 (0.0059)	0.9864 (0.0106)	0.9922 (0.0052)	0.9857 (0.0163)
Avg. of ρ^{Da} (S.D. of ρ^{Da})	0.9786 (0.0165)	0.9627 (0.0275)	0.7522 (0.1185)	0.6547 (0.1693)	0.6202 (0.1691)	0.6248 (0.1831)	0.7508 (0.1344)
Avg. of ρ^{Db} (S.D. of ρ^{Db})	0.3537 (0.1458)	0.2789 (0.1210)	0.1908 (0.1526)	0.7576 (0.1542)	-0.1123 (0.1453)	-0.1081 (0.1426)	-0.1159 (0.1401)
Avg. of ρ^{Ds} (S.D. of ρ^{Ds})	0.9944 (0.0024)	0.9971 (0.0016)	0.9452 (0.0258)	0.8760 (0.0586)	0.8872 (0.0503)	0.9487 (0.0339)	0.9724 (0.0300)
Avg. of ρ^{Dt} (S.D. of ρ^{Dt})	0.8486 (0.0408)	0.9382 (0.0323)	0.5387 (0.1099)	0.3619 (0.1408)	0.3018 (0.1537)	0.3013 (0.1950)	0.4506 (0.2231)
Avg. of ρ^{bend} (S.D. of ρ^{bend})	0.9945 (0.0008)	0.9992 (0.0005)	0.9980 (0.0010)	0.9969 (0.0037)	0.9941 (0.0071)	0.9955 (0.0040)	0.9876 (0.0179)
Avg. of ρ^{twist} (S.D. of ρ^{twist})	0.9594 (0.0125)	0.9975 (0.0014)	0.9549 (0.0163)	0.7047 (0.1299)	0.5984 (0.1337)	0.7538 (0.0824)	0.8492 (0.0678)
Avg. of ρ^c (S.D. of ρ^c)	0.9989 (0.0002)	0.9998 (0.0001)	0.9991 (0.0002)	0.9979 (0.0010)	0.9976 (0.0013)	0.9986 (0.0012)	0.9984 (0.0026)

Table S4

Average (Avg.) and standard deviation (S.D.) values of the correlations between the fluctuations of the 1PkN and 1P1N models for 500 randomly chosen $50 \times k$ bp sequences for $k = 9 \cdots 15$.

k	9	10	11	12	13	14	15
Avg. of ρ^a (S.D. of ρ^a)	0.9511 (0.0320)	0.9803 (0.0148)	0.9669 (0.0236)	0.9438 (0.0440)	0.8879 (0.0768)	0.9567 (0.0504)	0.9056 (0.0715)
Avg. of ρ^b (S.D. of ρ^b)	0.9394 (0.0454)	0.9711 (0.0283)	0.9671 (0.0238)	0.9468 (0.0386)	0.8618 (0.0955)	0.9503 (0.0531)	0.8704 (0.1038)
Avg. of ρ^s (S.D. of ρ^s)	0.9711 (0.0367)	0.9864 (0.0248)	0.9787 (0.0245)	0.9730 (0.0304)	0.9325 (0.0794)	0.9744 (0.0352)	0.9375 (0.0629)
Avg. of ρ^t (S.D. of ρ^t)	0.9274 (0.0451)	0.9681 (0.0272)	0.9439 (0.0384)	0.9379 (0.0406)	0.8473 (0.0947)	0.9461 (0.0528)	0.8610 (0.1087)
Avg. of ρ^{Da} (S.D. of ρ^{Da})	0.5660 (0.1972)	0.6076 (0.2031)	0.6095 (0.1955)	0.6629 (0.1772)	0.5824 (0.1949)	0.6156 (0.1961)	0.5972 (0.2023)
Avg. of ρ^{Db} (S.D. of ρ^{Db})	-0.0695 (0.1514)	-0.0411 (0.1533)	0.0039 (0.1463)	-0.0018 (0.1481)	0.0187 (0.1458)	0.0491 (0.1469)	0.0354 (0.1478)
Avg. of ρ^{Ds} (S.D. of ρ^{Ds})	0.8653 (0.0747)	0.9414 (0.0440)	0.9325 (0.0476)	0.8989 (0.0523)	0.7801 (0.1087)	0.8669 (0.0851)	0.7765 (0.1083)
Avg. of ρ^{Dt} (S.D. of ρ^{Dt})	0.1832 (0.3104)	0.2145 (0.3460)	0.2394 (0.3281)	0.3177 (0.3228)	0.2026 (0.3213)	0.2398 (0.3228)	0.2292 (0.2990)
Avg. of ρ^{bend} (S.D. of ρ^{bend})	0.9451 (0.0474)	0.9665 (0.0277)	0.9461 (0.0420)	0.9483 (0.0368)	0.8754 (0.0946)	0.9545 (0.0450)	0.8823 (0.0972)
Avg. of ρ^{twist} (S.D. of ρ^{twist})	-0.0028 (0.1566)	-0.0310 (0.1739)	-0.2316 (0.1536)	-0.1519 (0.1676)	-0.2917 (0.1578)	0.1388 (0.1785)	-0.1971 (0.1401)
Avg. of ρ^c (S.D. of ρ^c)	0.9870 (0.0094)	0.9948 (0.0043)	0.9929 (0.0046)	0.9913 (0.0053)	0.9712 (0.0207)	0.9898 (0.0121)	0.9740 (0.0220)

Table S5

Average (Avg.) and standard deviation (S.D.) values of the correlations between the fluctuations of the 1P3N and 1P1N models for 500 randomly chosen 450 bp sequences.

Avg. of ρ^a (S.D. of ρ^a)	0.9998 (0.0002)
Avg. of ρ^b (S.D. of ρ^b)	0.9998 (0.0001)
Avg. of ρ^s (S.D. of ρ^s)	0.9998 (0.0001)
Avg. of ρ^t (S.D. of ρ^t)	0.9998 (0.0001)
Avg. of ρ^{Da} (S.D. of ρ^{Da})	0.9231 (0.0514)
Avg. of ρ^{Db} (S.D. of ρ^{Db})	0.2769 (0.0691)
Avg. of ρ^{Ds} (S.D. of ρ^{Ds})	0.9972 (0.0022)
Avg. of ρ^{Dt} (S.D. of ρ^{Dt})	0.9180 (0.0560)
Avg. of ρ^{bend} (S.D. of ρ^{bend})	0.9998 (0.0001)
Avg. of ρ^{twist} (S.D. of ρ^{twist})	0.9992 (0.0005)
Avg. of ρ^c (S.D. of ρ^c)	0.9999 (0.0001)

SUPPLEMENTARY FIGURES

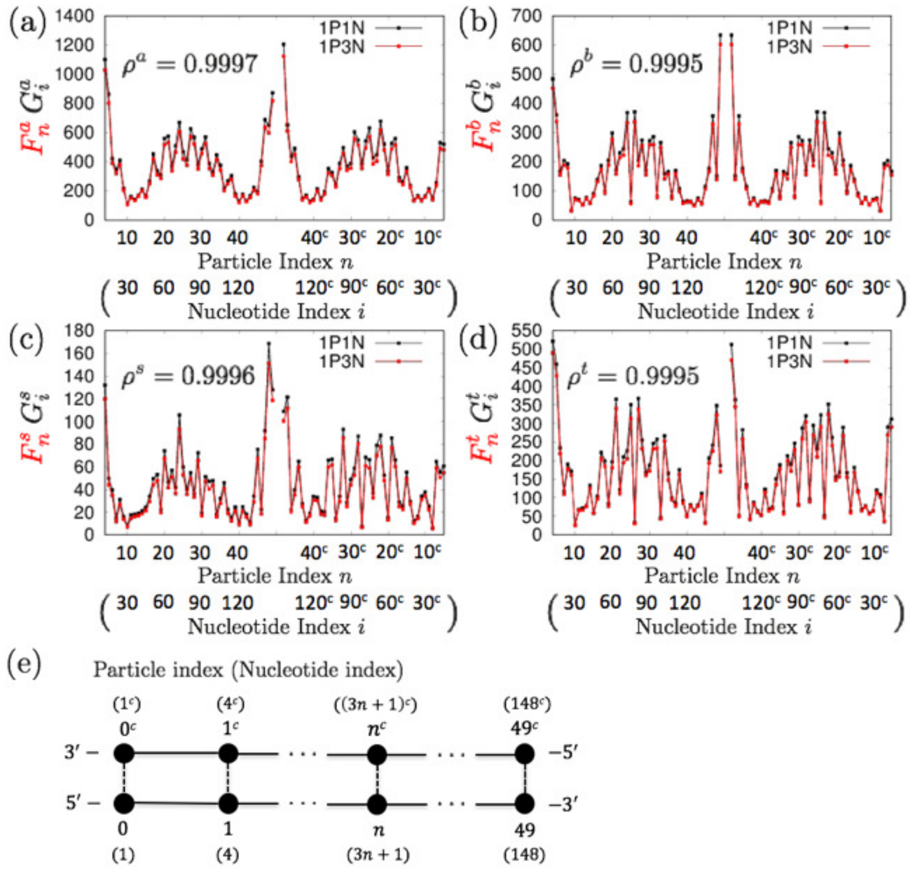

Figure S1. Comparisons of the fluctuations of particles between the 1P3N and 1P1N models for a typical 150-bp random sequence; (a) F_n^a and G_i^a; (b) F_n^b and G_i^b; (c) F_n^s and G_i^s; and (d) F_n^t and G_i^t. Black curves indicate the fluctuation profiles of the 1P1N model, and red curves show the fluctuation profiles of the 1P3N model. (e) Particle (nucleotide) indices.

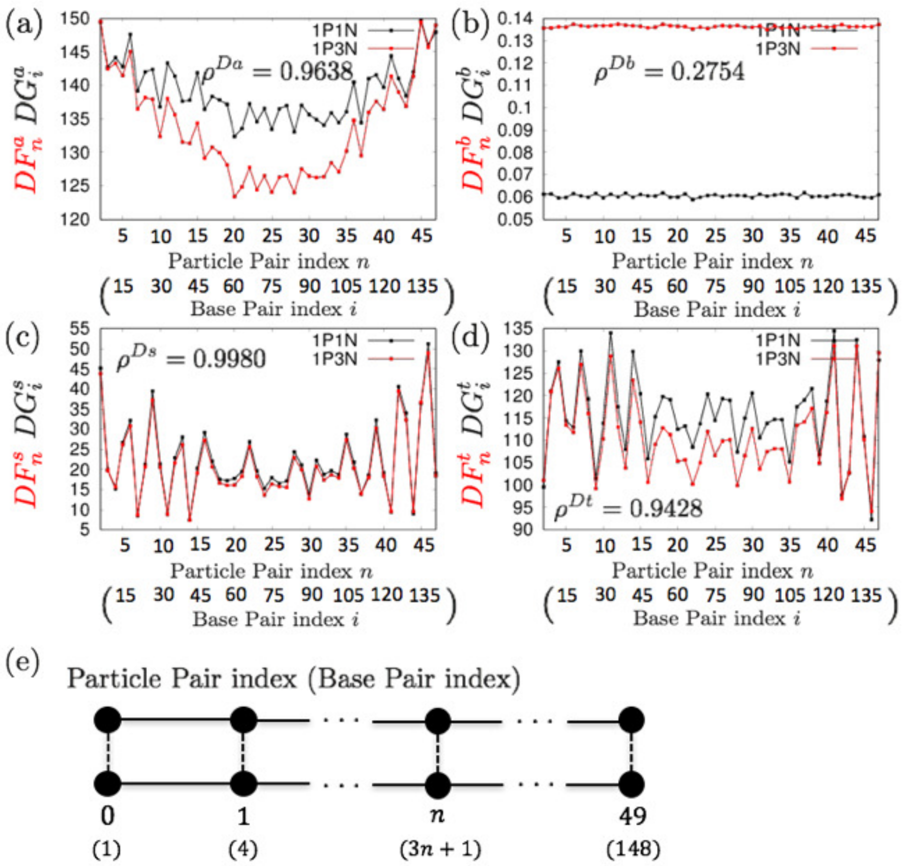

Figure S2. Comparisons of the fluctuations of particle pairs between the 1P3N and 1P1N models for a typical 150-bp random sequence for (a) DF_n^a and DG_i^a; (b) DF_n^b and DG_i^b; (c) DF_n^s and DG_i^s; and (d) DF_n^t and DG_i^t. Black curves indicate the fluctuation profiles of the 1P1N model, and red curves show the fluctuation profiles of the 1P3N model. (e) Particle pair (base pair) indices.

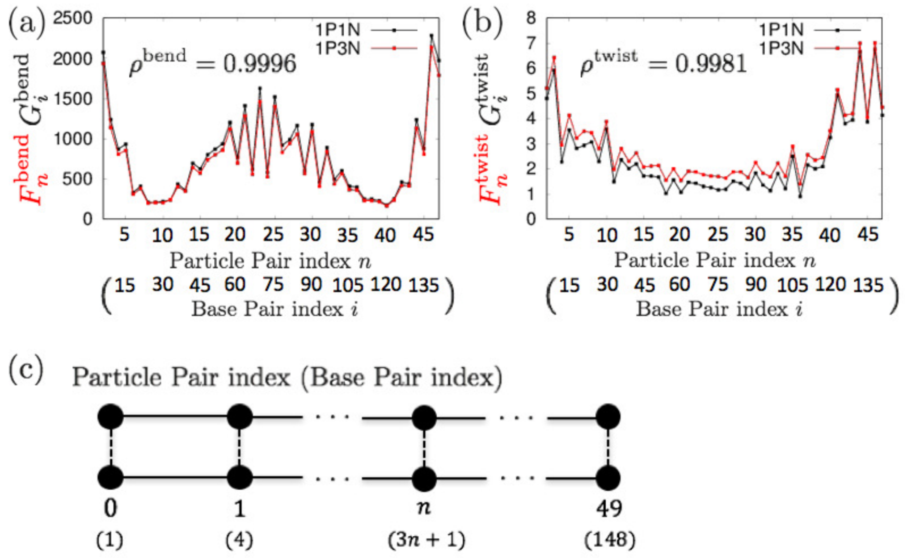

Figure S3. Comparisons of the fluctuations of particle pairs between the 1P3N and 1P1N models for a typical 150-bp random sequence for (a) F_n^{bend} and G_i^{bend}; (b) F_n^{twist} and G_i^{twist}. Black curves indicate the fluctuation profiles of the 1P1N model, and red curves show the fluctuation profiles of the 1P3N model. (c) Particle pair (base pair) indices.

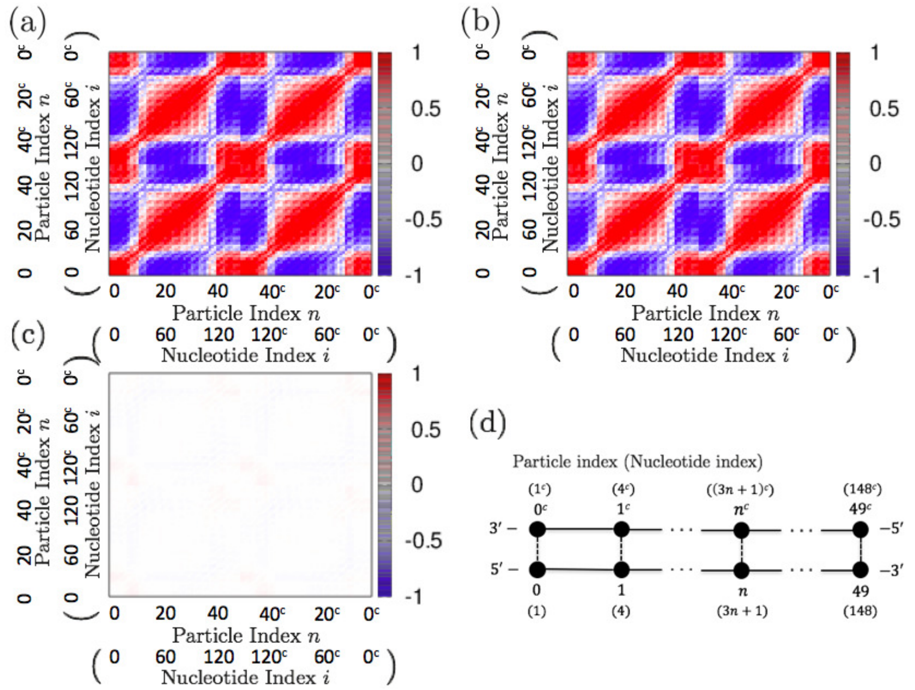

Figure S4. Comparisons between G_{ij}^c of the 1P1N model and F_{nl}^c of the 1P3N model ($i = 3n + 1$, $j = 3l + 1$), for a typical 150-bp sequence; (a) G_{ij}^c; (b) F_{nl}^c; (c) $G_{ij}^c - F_{nl}^c$; and $\rho^c = 0.9999$. (d) Particle (nucleotide) indices.

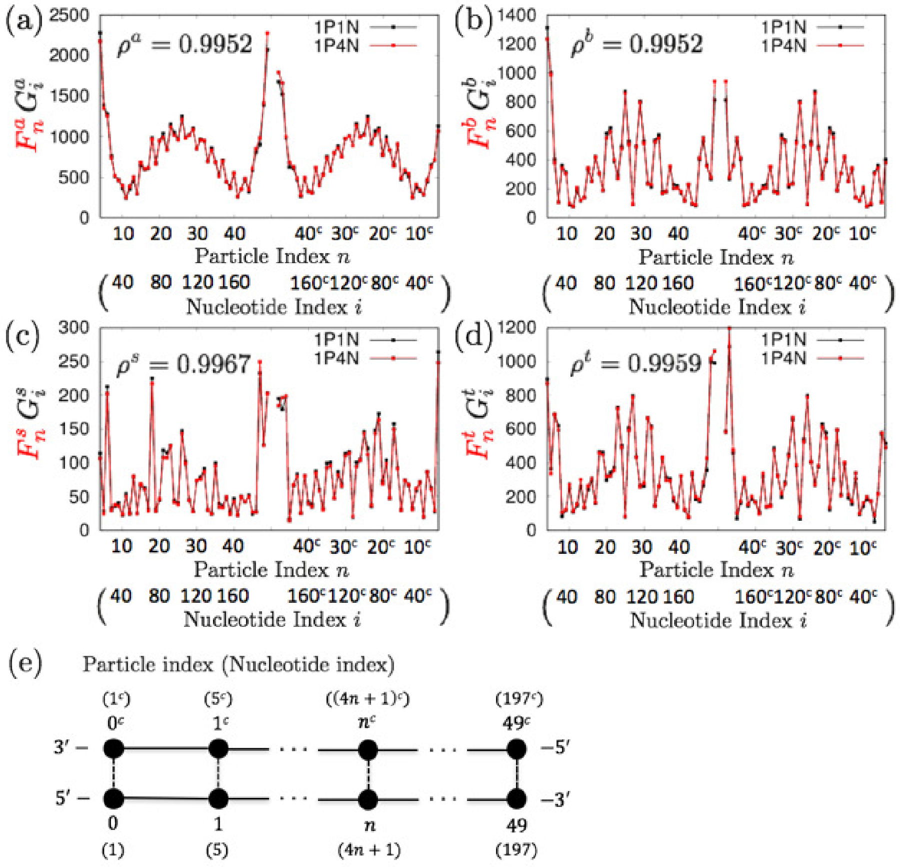

Figure S5. Comparisons of the fluctuations of particles between the 1P4N and 1P1N models for a typical 200-bp random sequence; (a) F_n^a and G_i^a; (b) F_n^b and G_i^b; (c) F_n^s and G_i^s; and (d) F_n^t and G_i^t. Black curves show the fluctuation profiles of the 1P1N model, and red curves show those of the 1P4N model. (e) Particle (nucleotide) indices.

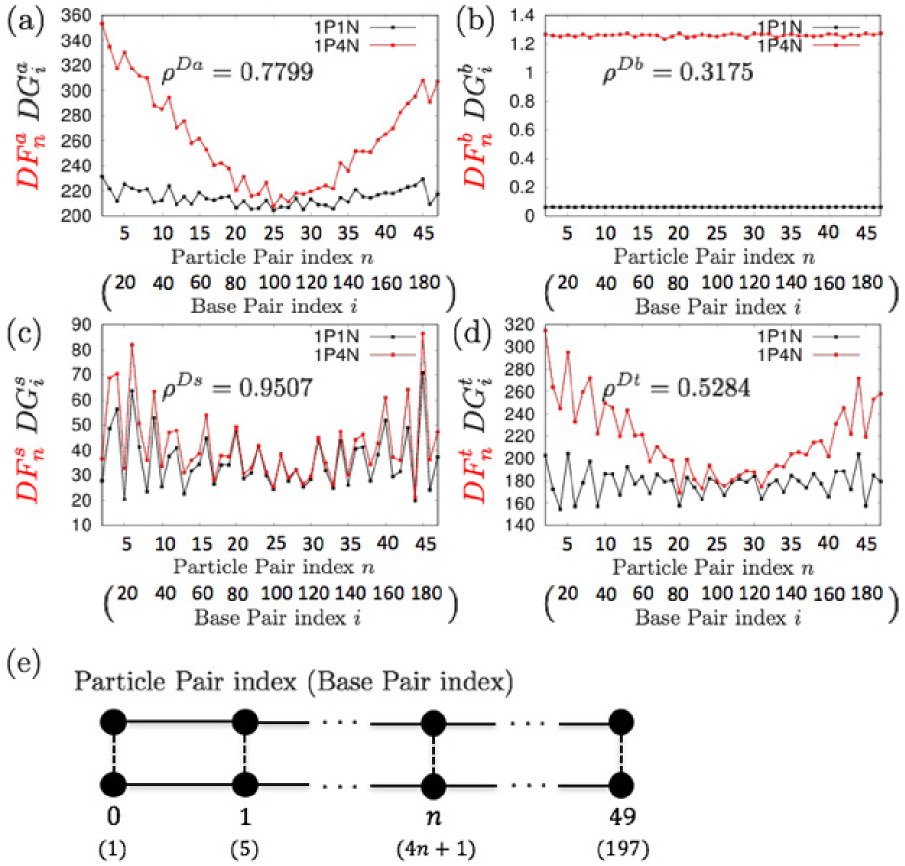

Figure S6. Comparisons of the fluctuations of particle pairs between the 1P4N and 1P1N models for a typical 200-bp random sequence for (a) DF_n^a and DG_i^a; (b) DF_n^b and DG_i^b; (c) DF_n^s and DG_i^s; and (d) DF_n^t and DG_i^t. Black curves show the fluctuation profiles of the 1P1N model, and red curves show those of the 1P4N model. (e) Particle pair (base pair) indices.

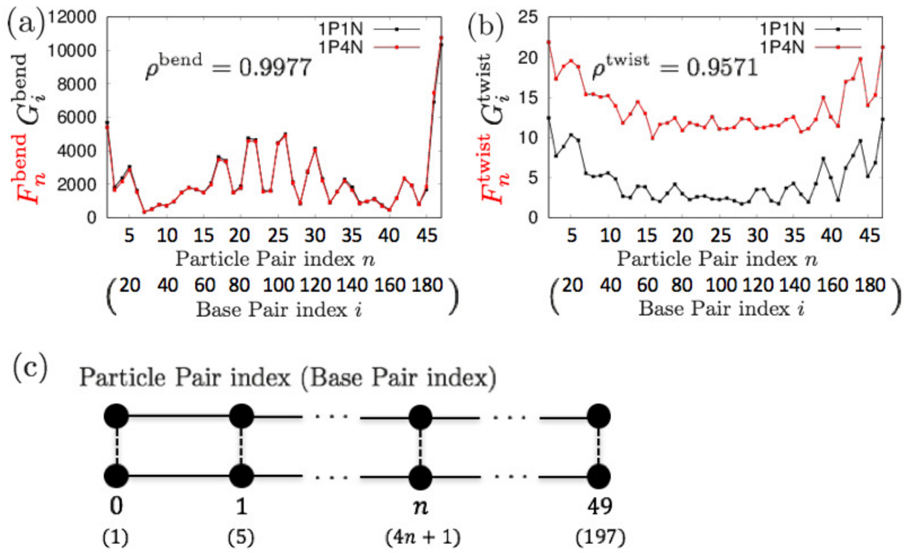

Figure S7. Comparisons of the fluctuations of particle pairs between the 1P4N and 1P1N models for a typical 200-bp random sequence for (a) F_n^{bend} and G_i^{bend}; (b) F_n^{twist} and G_i^{twist}. Black curves show the fluctuation profiles of the 1P1N model, and red curves show those of the 1P4N model. (c) Particle pair (base pair) indices.

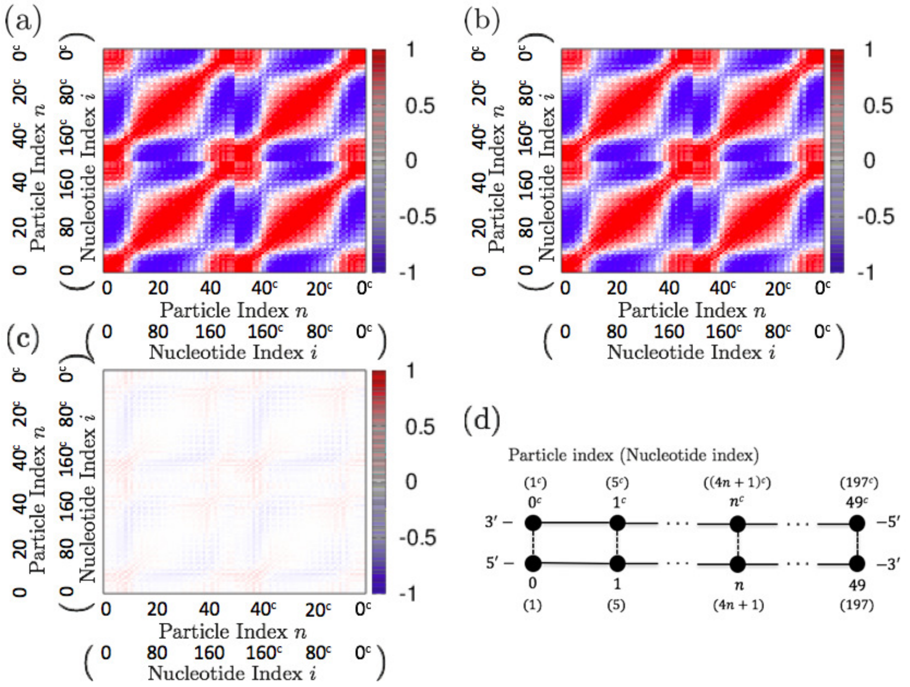

Figure S8. Comparisons between G^c_{ij} of the 1P1N model and F^c_{nl} of the 1P4N model ($i = 4n + 1$, $j = 4l + 1$), for a typical 200-bp sequence. (a) G^c_{ij}; (b) F^c_{nl}; (c) $G^c_{ij} - F^c_{nl}$; and $\rho^c = 0.9990$. (d) Particle (nucleotide) indices.

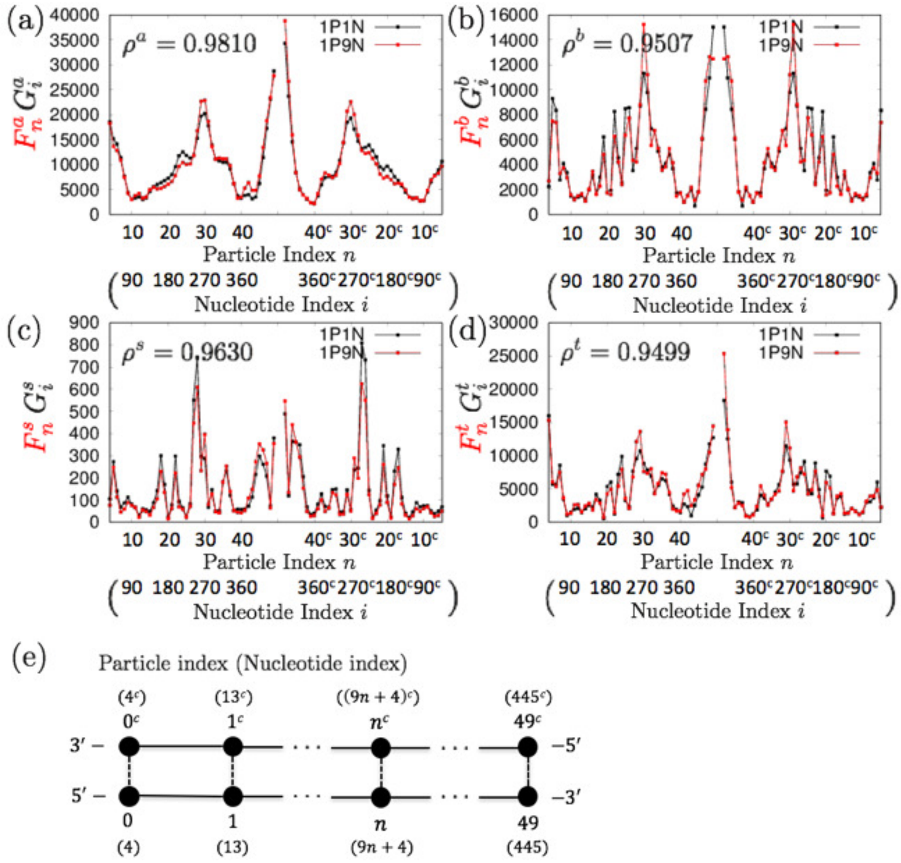

Figure S9. Comparisons of the fluctuations of particles between the 1P9N and 1P1N models for a typical 450-bp random sequence; (a) F_n^a and G_i^a; (b) F_n^b and G_i^b; (c) F_n^s and G_i^s; and (d) F_n^t and G_i^t. Black curves show the fluctuation profiles of the 1P1N model, and red curves show those of the 1P9N model. (e) Particle (nucleotide) indices.

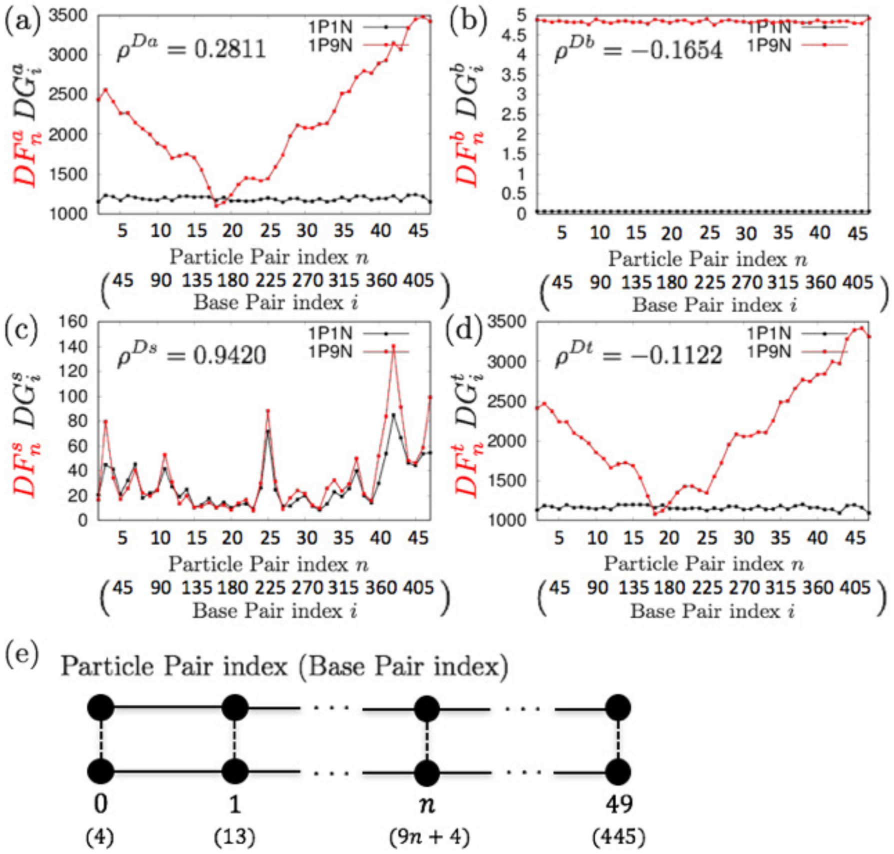

Figure S10. Comparisons of the fluctuations of particle pairs between the 1P9N and 1P1N models for a typical 450-bp random sequence for (a) DF_n^a and DG_i^a; (b) DF_n^b and DG_i^b; (c) DF_n^s and DG_i^s; and (d) DF_n^t and DG_i^t. Black curves show the fluctuation profiles of the 1P1N model, and red curves show those of the 1P9N model. (e) Particle pair (base pair) indices.

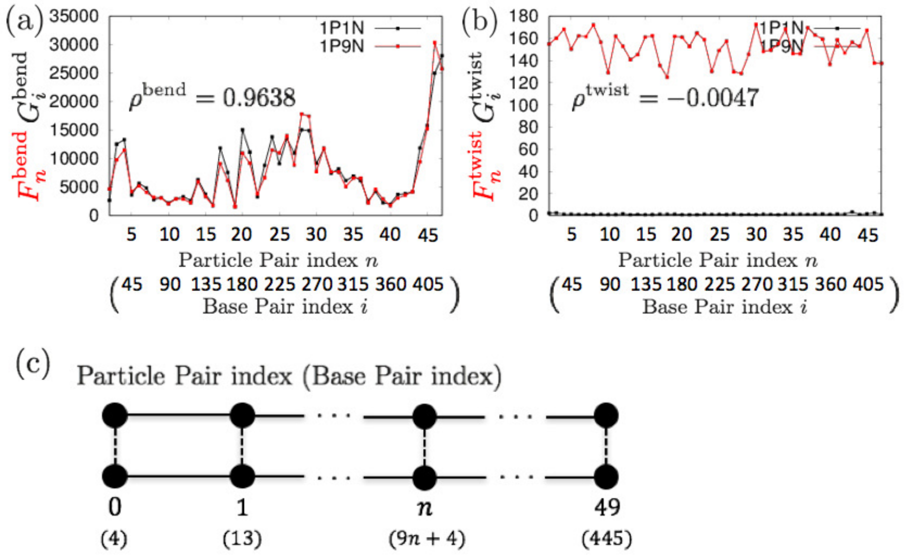

Figure S11. Comparisons of the fluctuations of particle pairs between the 1P9N and 1P1N models for a typical 450-bp random sequence for (a) F_n^{bend} and G_i^{bend}; (b) F_n^{twist} and G_i^{twist}. Black curves show the fluctuation profiles of the 1P1N model, and red curves show those of the 1P9N model. (c) Particle pair (base pair) indices.

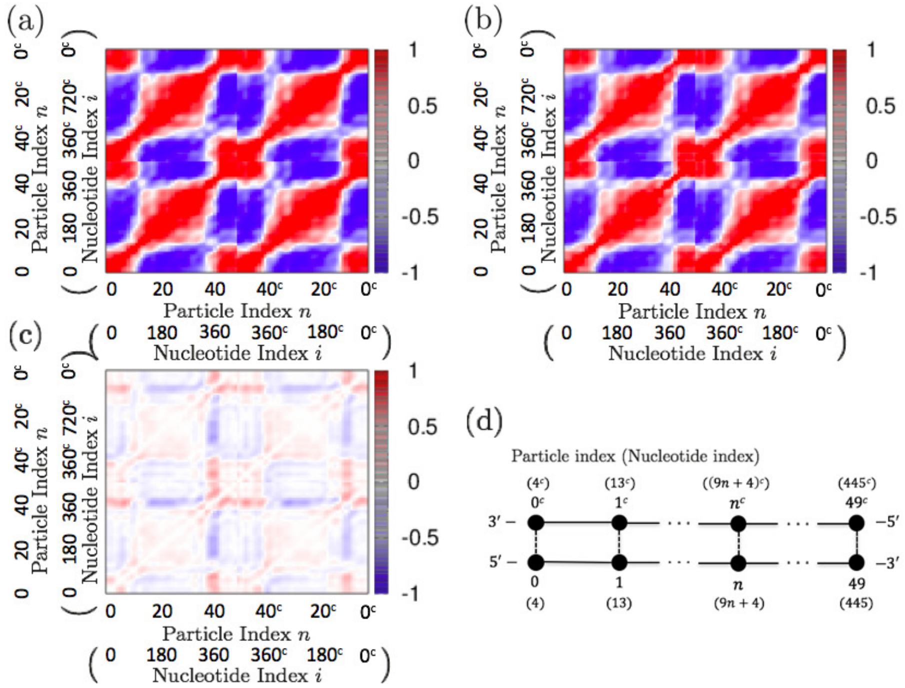

Figure S12. Comparisons between G_{ij}^c of the 1P1N model and F_{nl}^c of the 1P9N model ($i = 9n + 4$, $j = 9l + 4$), for a typical 450-bp sequence. (a) G_{ij}^c; (b) F_{nl}^c; (c) $G_{ij}^c - F_{nl}^c$; and $\rho^c = 0.9934$. (d) Particle (nucleotide) indices.

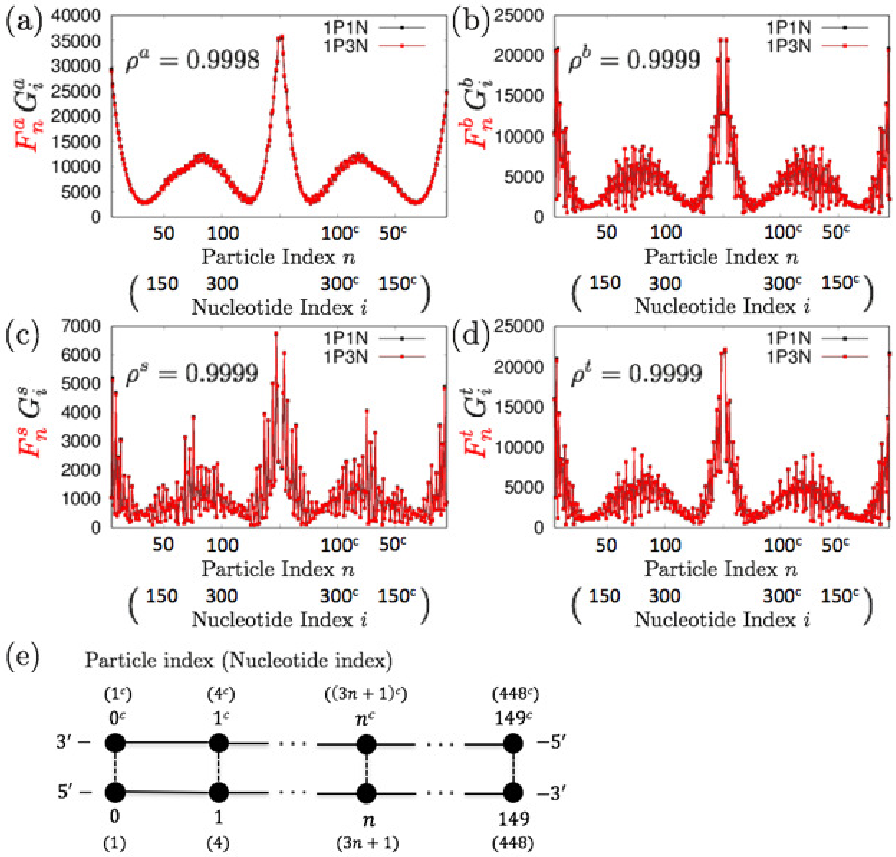

Figure S13. Comparisons of the fluctuations of particles between the 1P3N and 1P1N models for a typical 450-bp random sequence; (a) F_n^a and G_i^a; (b) F_n^b and G_i^b; (c) F_n^s and G_i^s; and (d) F_n^t and G_i^t. Black curves indicate the fluctuation profiles of the 1P1N model, and red curves show the fluctuation profiles of the 1P3N model. (e) Particle (nucleotide) indices.

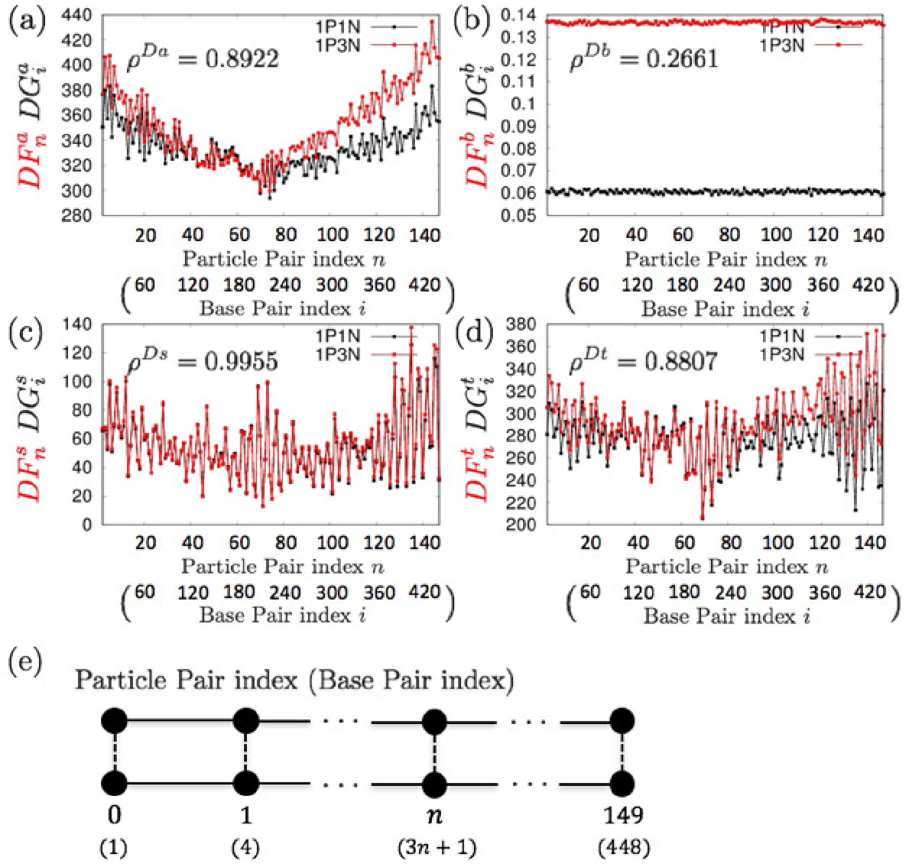

Figure S14. Comparisons of the fluctuations of particle pairs between the 1P3N and 1P1N models for a typical 450-bp random sequence for (a) DF_n^a and DG_i^a; (b) DF_n^b and DG_i^b; (c) DF_n^s and DG_i^s; and (d) DF_n^t and DG_i^t. Black curves indicate the fluctuation profiles of the 1P1N model, and red curves show the fluctuation profiles of the 1P3N model. (e) Particle pair (base pair) indices.

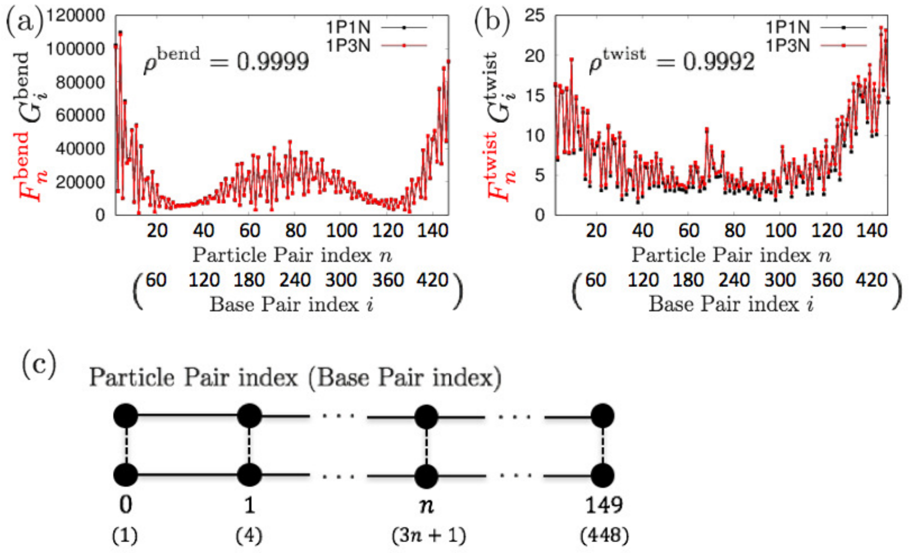

Figure S15. Comparisons of the fluctuations of particle pairs between the 1P3N and 1P1N models for a typical 450-bp random sequence for (a) F_n^{bend} and G_i^{bend}; (b) F_n^{twist} and G_i^{twist}. Black curves indicate the fluctuation profiles of the 1P1N model, and red curves show the fluctuation profiles of the 1P3N model. (c) Particle pair (base pair) indices.

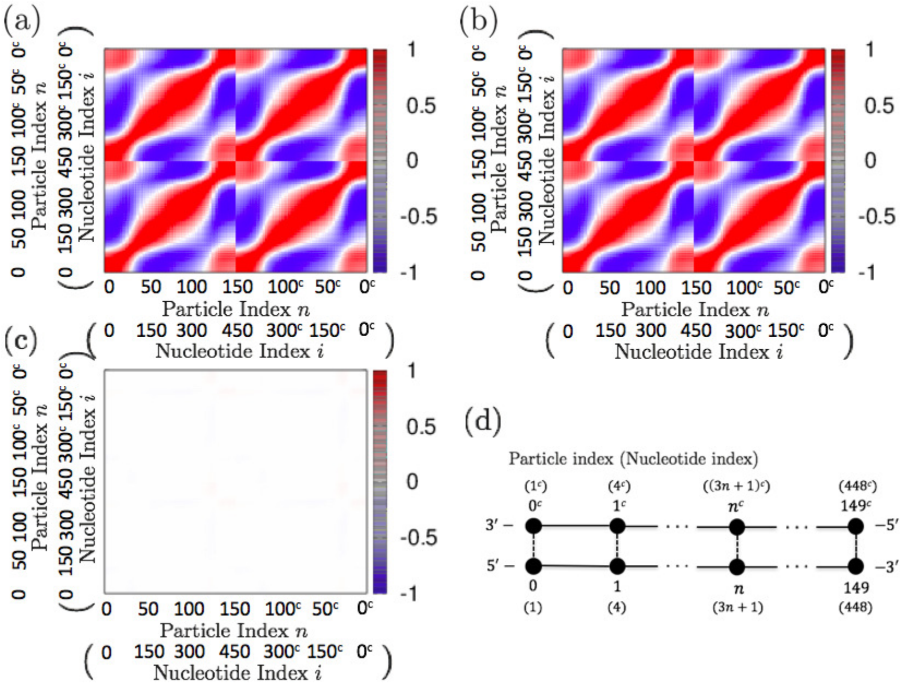

Figure S16. Comparisons between G_{ij}^c of the 1P1N model and F_{nl}^c of the 1P3N model ($i = 3n + 1$, $j = 3l + 1$), for a typical 450-bp sequence. (a) G_{ij}^c; (b) F_{nl}^c; (c) $G_{ij}^c - F_{nl}^c$; and $\rho^c = 0.9999$. (d) Particle (nucleotide) indices.

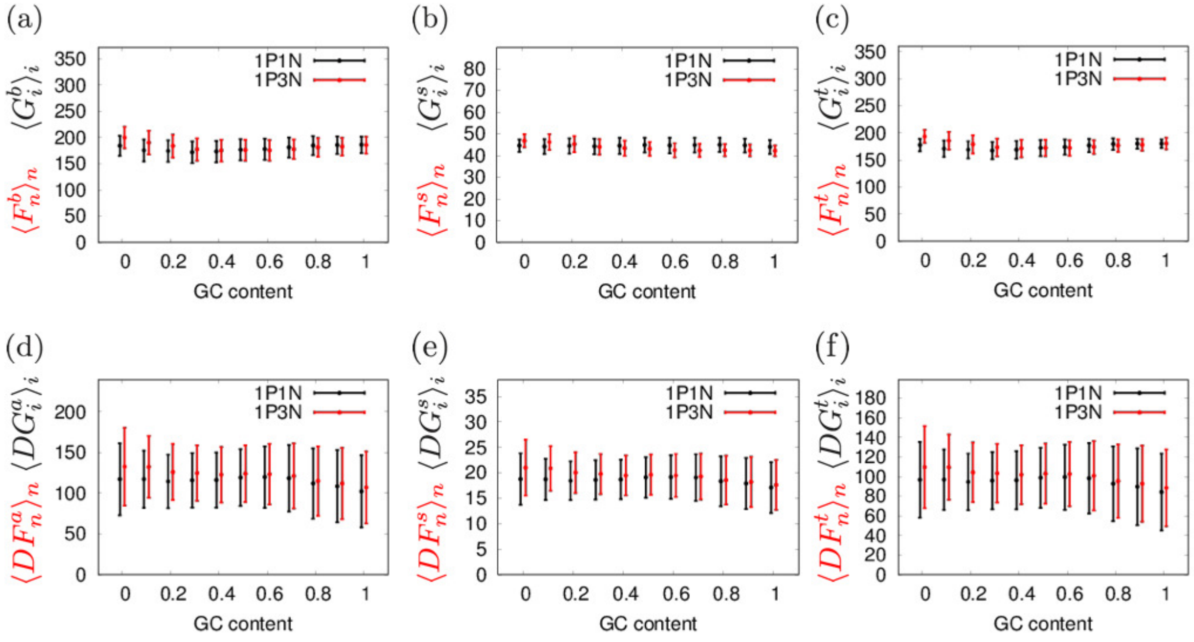

Figure S17. Average ± standard deviations of (a) $\langle F_n^b \rangle_n$ and $\langle G_i^b \rangle_i$, (b) $\langle F_n^s \rangle_n$ and $\langle G_i^s \rangle_i$, (c) $\langle F_n^t \rangle_n$ and $\langle G_i^t \rangle_i$, (d) $\langle DF_n^a \rangle_n$ and $\langle DG_i^a \rangle_i$, (e) $\langle DF_n^s \rangle_n$ and $\langle DG_i^s \rangle_i$, and (f) $\langle DF_n^t \rangle_n$ and $\langle DG_i^t \rangle_i$ for 1000 samples of random 150-bp sequences with an average GC-content = 0, 0.1, 0.2, $\cdots$ 1.

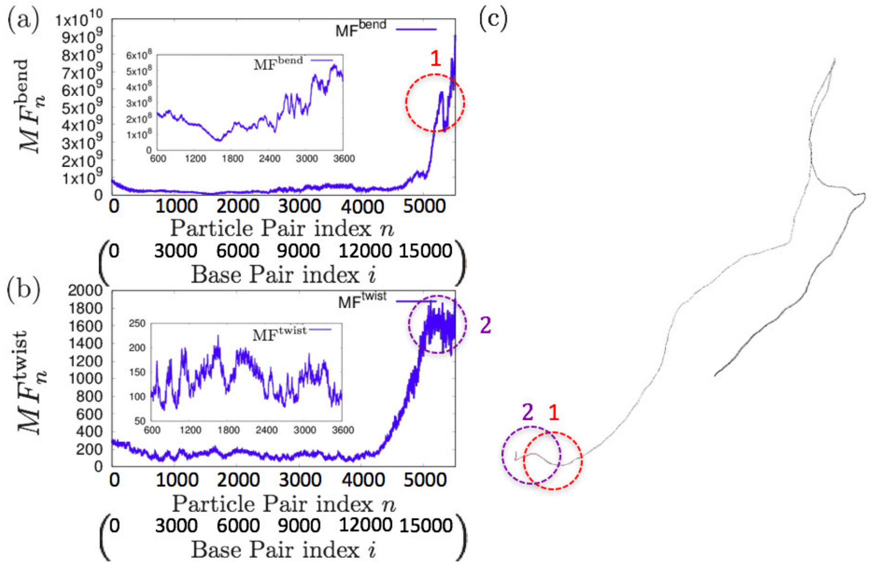

Figure S18. Fluctuations of the n-th particle pair (i-th base pair) and the basic structure of human mitochondrial DNA in the case of an unknown base assumed to be guanine. (a) Distribution of bending fluctuations. (b) Distribution of twisting fluctuations. (c) Basic structure of the analyzed genome and the corresponding regions analyzed in (a) and (b).

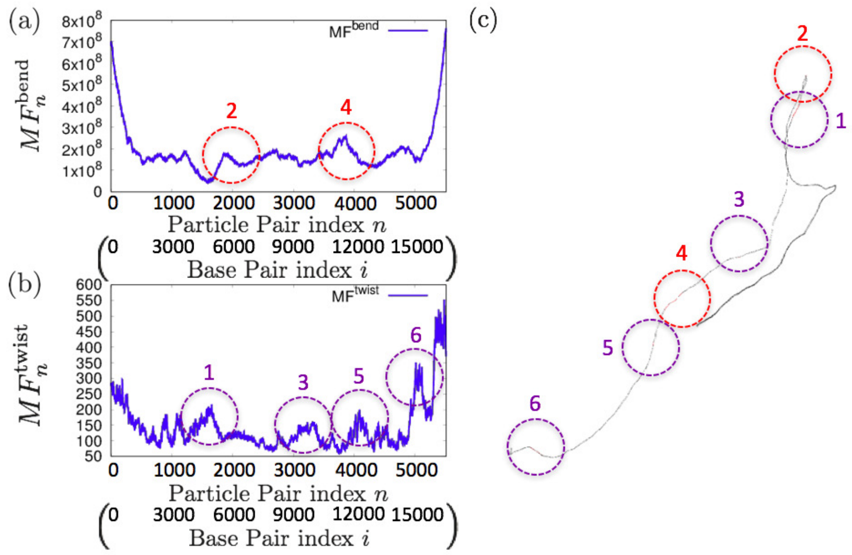

Figure S19. Fluctuations of the n-th particle pair (i-th base pair) and the basic structure of human mitochondrial DNA in the case of an unknown base assumed to be cytosine. (a) Distribution of bending fluctuations. (b) Distribution of twisting fluctuations. (c) Basic structure of the analyzed genome and the corresponding regions analyzed in (a) and (b).

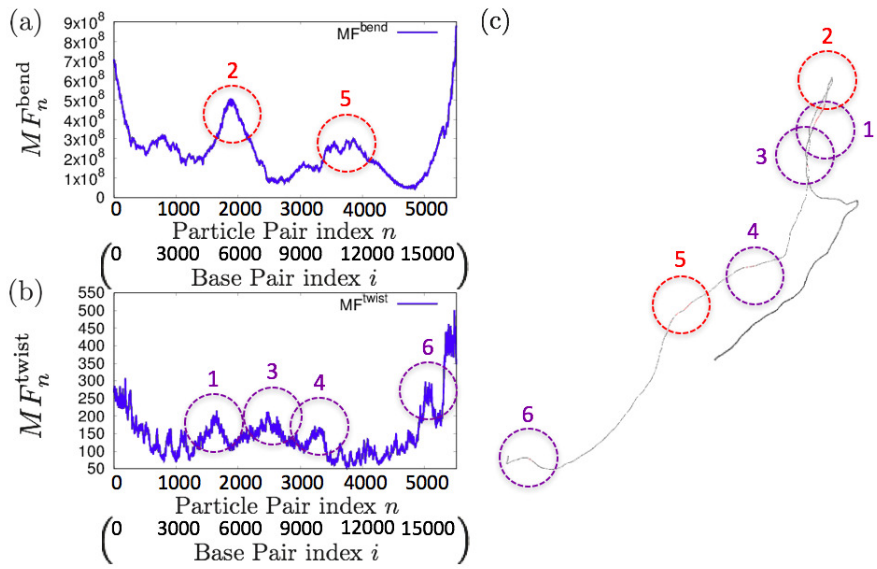

Figure S20. Fluctuations of the n-th particle pair (i-th base pair) and the basic structure of human mitochondrial DNA in the case of an unknown base assumed to be thymine. (a) Distribution of bending fluctuations. (b) Distribution of twisting fluctuations. (c) Basic structure of the analyzed genome and the corresponding regions analyzed in (a) and (b).

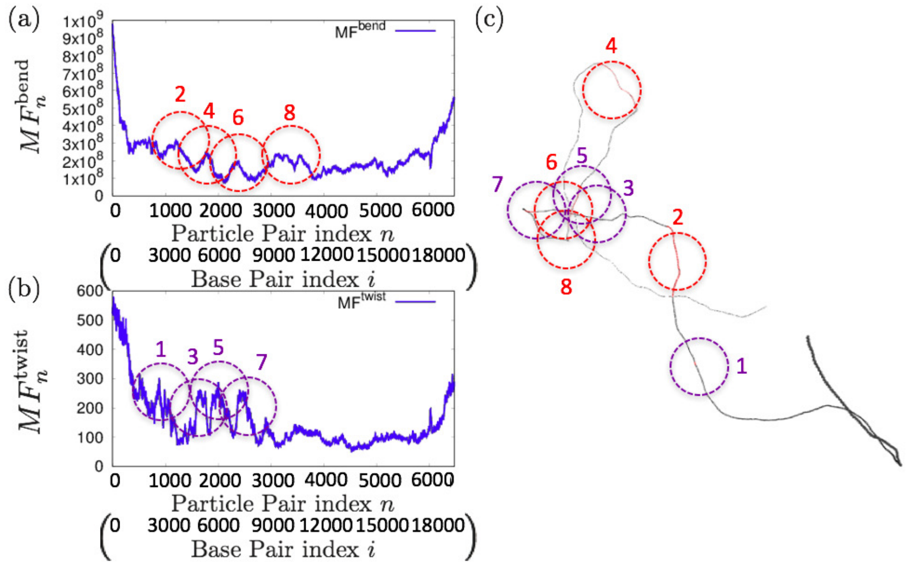

Figure S21. Fluctuations of the n-th particle pair (i-th base pair) and the basic structure of *Schizosaccharomyces pombe* mitochondrial DNA. (a) Distribution of bending fluctuations. (b) Distribution of twisting fluctuations. (c) Basic structure of the analyzed genome and the corresponding regions analyzed in (a) and (b).

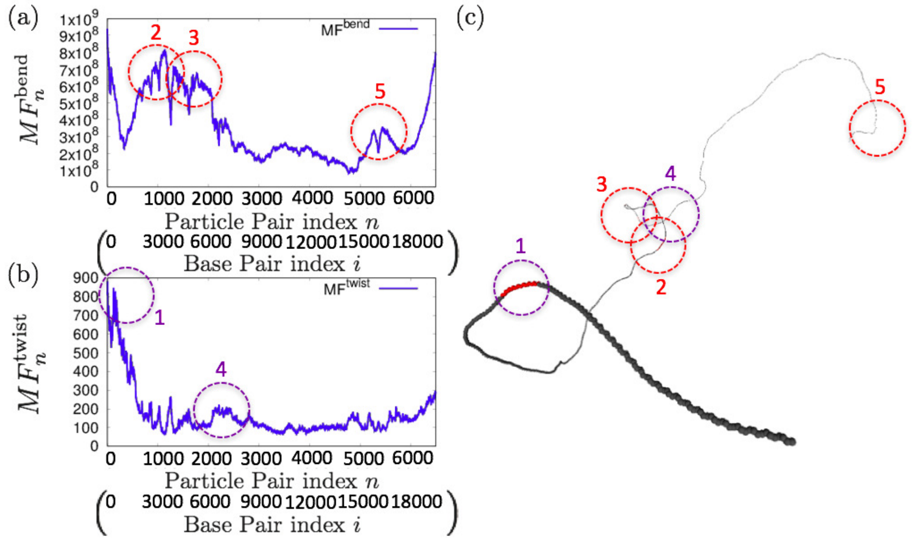

Figure S22. Fluctuations of the n-th particle pair (i-th base pair) and the basic structure of *Drosophila melanogaster* mitochondrial DNA. (a) Distribution of bending fluctuations. (b) Distribution of twisting fluctuations. (c) Basic structure of the analyzed genome and the corresponding regions analyzed in (a) and (b).
